# The Electronic Effects of 3-Methoxycarbonylcoumarin Substituents on Spectral, Antioxidant, and Protein Binding Properties

**DOI:** 10.3390/ijms241411820

**Published:** 2023-07-23

**Authors:** Jelena Vasić, Dušan Dimić, Marko Antonijević, Edina H. Avdović, Dejan Milenković, Đura Nakarada, Jasmina Dimitrić Marković, Maja Molnar, Melita Lončarić, Drago Bešlo, Zoran Marković

**Affiliations:** 1 Faculty of Physical Chemistry, University of Belgrade, Studentski trg 12-16, 11000 Belgrade, Serbia; 2 Institute for Information Technologies Kragujevac, University of Kragujevac, Jovana Cvijica bb, 34000 Kragujevac, Serbia; 3 Department of Applied Chemistry and Ecology, Faculty of Food Technology Osijek, Josip Juraj Strossmayer University of Osijek, Franje Kuhača 18, 31000 Osijek, Croatiamelita2608@gmail.com (M.L.); 4 Department of Agroecology and Environmental Protection, Faculty of Agrobiotechnical Sciences Osijek, Josip Juraj Strossmayer University of Osijek, Vladimir Prelog 1, 31000 Osijek, Croatia

**Keywords:** coumarin, BSA, DFT, EPR, molecular dynamics

## Abstract

Coumarin derivatives are a class of compounds with pronounced biological activities that depend primarily on the present substituents. Four 3-methoxycarbonylcoumarin derivatives with substituents of different electron-donating/electron-withdrawing abilities (Br, NO_2_, OH, and OMe) were investigated structurally by NMR, IR, and UV-VIS spectroscopies and density functional theory methods. The appropriate level of theory (B3LYP-D3BJ/6-311++G(d,p) was selected after comparing similar compounds’ experimental and theoretical structural parameters. The natural bond orbital and quantum theory of atoms in molecules were employed to investigate the intramolecular interactions governing stability. The electronic effects of substituents mostly affected the aromatic ring that the substituents are directly attached to. The antioxidant properties were investigated by electron paramagnetic resonance spectroscopy towards HO^•^, and the percentages of reduction were between 13% (**6-Br**) and 23% (**6-OMe**). The protein binding properties towards transport proteins were assessed by spectrofluorimetry, molecular docking, and molecular dynamics (MD). The experimentally determined binding energies were well reproduced by molecular docking, showing that the spontaneity of ibuprofen binding was comparable to the investigated compounds. The flexibility of HSA in MD simulations depended on the substituents. These results proved the importance of electronic effects for the protein binding affinities and antioxidant properties of coumarin derivatives.

## 1. Introduction

**Figure 1:**
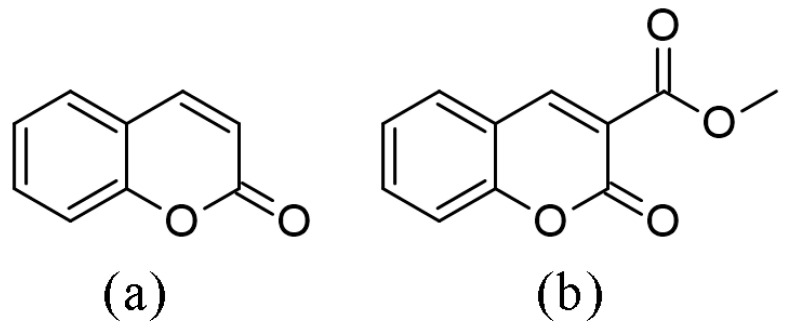
Structures of (**a**) coumarin and (**b**) 3-methoxycarbonylcoumarin.

Coumarin and its derivates belong to the large benzopyrone family, structurally characterized by the benzene ring joined to a pyrone ring (Figure 1a). This simple structure is the parent molecule of all coumarins and represents the base of much more complex compounds [1]. Coumarins and their derivatives are naturally distributed in many plants’ seeds, roots, leaves, and fruits (e.g., blueberries, green tea, and essential oils) [2]. In plants, coumarins are involved in the processes of growth and photosynthesis [3]. Microbial sources of coumarins are also described in the literature [4]. Some coumarins, such as novobiocin and coumermycin, are obtained from *Streptomyces* species and aflatoxins from *Aspergillus* species. Coumermycin, structurally similar to novobiocin, is nearly 50 times more potent than novobiocin against *Escherichia coli* and *Staphylococcus aureus*. Coumermycin also inhibits the supercoiling of DNA catalyzed by *Escherichia coli* DNA gyrase [5].

Coumarins have several attractive characteristics, such as low molecular weight, simple structure, high bioavailability, and high solubility in most organic solvents and oils, which, together with their multiple biological activities, ensure a prominent role in drug research and development. The extraction, synthesis, and evaluation of coumarins have become a rapidly developing topic because of their beneficial effects on human health, such as reducing the risk of cancer, diabetes, and cardiovascular and brain diseases [6]. Their antitumor [7,8,9,10], photochemotherapy, anti-HIV [11], antibacterial and antifungal [12,13,14], anti-inflammatory [15,16], anticoagulant effects, and central nervous system stimulant effects [17] make coumarins interesting in modern medicinal chemistry. A potent antioxidant and protective effect against oxidative stress by scavenging the reactive oxygen species has also been reported for hydroxycoumarins [18,19,20]. In addition, the discovery of coumarins with weak estrogenic activity included this type of compound in the prevention of menopausal distress [2]. Coumarin removes protein and edema fluid from injured tissue by stimulating phagocytosis and enzyme production [21].

The electronic effects of substituents influence the stability and reactivity of coumarin derivatives, as well as many of the biological and pharmacological properties [22,23,24,25], such as lipophilicity, bioavailability [26], radical scavenging activity, and acetylcholinesterase inhibition [27]. Therefore, the electron donating/withdrawing effects of four common substituents (Br, NO_2_, OH, and OMe) on 3-methoxycarbonylcoumarins’ structure (Figure 1b), NMR, IR, UV-VIS, antioxidant, and protein binding activity are investigated experimentally and theoretically in this contribution. Density functional theory was applied to predict, compare, and assign the mentioned spectra with the experimental ones. The antioxidant activity of these derivatives towards the hydroxyl radical, a biologically relevant free radical, was investigated by electron paramagnetic resonance spectroscopy. The binding of selected derivatives towards bovine serum albumin was analyzed by spectrofluorimetry. Additionally, molecular docking and molecular dynamics studies were used to access the binding mode at the molecular level and to quantify the possible interactions.

## 2. Results and Discussion

### 2.1. Structural Optimization, NBO, and QTAIM Analysis

**Figure 2:**
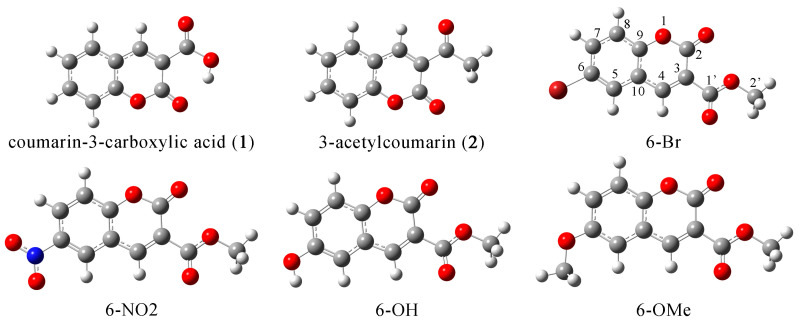
Optimized structures of coumarin derivatives at the B3LYP-D3BJ/6-311++G(d,p) level of theory.

The selection of an appropriate DFT level of theory is often based on comparing optimized and crystallographic structures. As the experimental data are unavailable for the investigated compounds, the authors have selected two structurally similar molecules from the Cambridge crystallographic data center, namely coumarin-3-carboxylic acid (**1**) and 3-acetylcoumarin (**2**) (Figure 2). These compounds have the same core but differ in the present substituents from the investigated coumarin derivatives. Three common functionals (B3LYP-D3BJ, M06-2X, and APFD) were selected for optimization, as these provided satisfactory results in previous studies on similar compounds [28,29]. Two parameters were used to compare experimental and theoretical data, the correlation coefficient (R) and mean absolute error (MAE) (Table 1). The latter represents an average value of the absolute differences between the values of two data sets. The experimental and optimized bond lengths and angles for both compounds are shown in Tables S1–S4.

The results shown in Table 1 prove that the selected functionals optimize the crystallographic structure well. This can be expected as the chosen structures were rigid with extended delocalization throughout a major part of a molecule. The correlation coefficients for bond lengths of **1** obtained by three common functionals are almost equal, while in the case of **2,** the values range from 0.995 (APFD) to 0.998 (M06-2X). The MAE values are between 0.014 and 0.15 Å for **1** and between 0.006 and 0.007 Å for **2**. Based on these values, a clear distinction between functionals’ performances cannot be made, and bond angles were used further for the selection. The values of R in the case of **1** are similar and range between 0.936 (M06-2X) and 0.945 (APFD), while the same parameter for **2** is between 0.979 (M06-2X) and 0.983 (B3LYP-D3BJ). The lowest MAE values for bond angles were calculated for B3LYP-D3BJ. Therefore, the B3LYP-D3BJ/6-311++G(d,p) theory level was selected to optimize coumarin derivatives. The same level of theory was previously successfully applied for the spectral assignation and reactivity investigation of similar systems [29,30,31,32].

Four coumarin derivatives containing different substituents were optimized at the mentioned level of theory, and their structures are presented in Figure 2. These derivatives contain substituents with various electron-donating/electron-withdrawing effects. The bond lengths and angles are provided in Tables S5 and S6. The investigated coumarin structures are planar with elongated delocalization through an aromatic ring and strong donation from present carbonyl groups; therefore, a significant influence of substituents to structural parameters is not expected. This was proven when bond lengths for the common parts of molecules were compared (to **6-Br**) and the MAE values were calculated. These values were 0.002 (**6-OH**) and 0.003 Å (**6-NO_2_** and **6-OMe**), equal to the experimental uncertainty of the crystallographic analysis. The same applies to the bond angles with MAEs equal to 0.2 (**6-NO_2_** and **6-OH**) and 0.3° (**6-OMe**). The bond distance between carbon and heteroatom of the substituent is the highest in the case of **6-Br** (1.915 Å). When OH and OMe groups are present, the bond lengths are almost equal, namely 1.365 (**6-OH**) and 1.359 Å (**6-OMe**). The planarity of substituents with the aromatic ring is preserved in the case of all substituents, with the angles on both sides being almost equal, which allows easy electron donation through positive resonant effect (in the case of Br, OH, and OMe) or electron withdrawal (in case of NO_2_). These effects are additionally investigated within NBO and QTAIM analyses in the following paragraph.

The intramolecular interactions governing the stability of compounds can be quantified through the second-order perturbation theory. Some of the most prominent stabilization interactions are listed in Table S7. Due to the presence of aromatic rings and carbonyl groups within a structure, four derivatives share some common stabilization interactions. The most numerous stabilization interactions are formed within an aromatic ring, denoted as π(C-C)→π*(C-O1), with a range of energies between 55 and 150 kJ mol^−1^ (Table S8). This type of interaction with similar strength is present in the pyrone ring. This ring is additionally stabilized by the interactions between groups, including oxygen atoms. In these interactions, the donating group can be C=C through π(C3-C4)→π*(C2-O) interaction with energies between 97 and 109 kJ mol^−1^. The lone pair on the oxygen atom can be a donating group through LP(O)→π*(C2-O) and LP(O)→π*(C9-C10), with energies higher than 120 kJ mol^−1^ in most cases. The carbonyl oxygen of the pyrone ring can also act as donating group with similar interaction energies. High stabilization energies were also obtained in the ester group of the substituent. As expected, the interactions within the ester group had stabilization energies of 120 kJ mol^−1^. These oxygen atoms also stabilize the C4−C10 bond that connects the substituent and pyrone ring. Based on these values, it can be concluded that the coumarin core is very stable due to the extended delocalization between groups. Similar results were observed for other coumarin derivatives [12,29]. The main difference in the stability of the four derivatives comes from the stabilization interactions that include substituents. The neighboring C−C bonds interact with the C−substituent bond. These interactions can be weak with stabilization energy of only 5 kJ mol^−1^ in the case of the electron-withdrawing NO_2_ group (Table S7). Stronger interactions were observed in the case of a weak electron donor, Br, with stabilization energies of 12 kJ mol^−1^. An oxygen atom in OH and OMe groups acts as a strong electron-donor, and these stabilization interactions have energies between 23 and 42 kJ mol^−1^. Bromine also interacts through a lone pair with neighboring bonds, LP(Br)→π*(C5-C6) (40 kJ mol^−1^) and LP(Br)→π*(C6-C7) (14 kJ mol^−1^). Nitrogen and oxygen atoms of the NO_2_ group additionally stabilize other groups within a nitro group through strong interactions, such as LP(O)→π*(N-O) (540 kJ mol^−1^). Once OH and OMe are present as substituents, donation from substituents to the rest of the molecules increases. These energy values prove the assumption that extended delocalization is enhanced strongly in the case of the last two groups but also explain the co-planarity of all substituents with an aromatic ring, observed in the previous section.

QTAIM analysis was performed to investigate the effect of substituents on the neighboring groups. As it was previously shown that these substituents do not significantly affect groups that are further away, they are not included in the discussion. Table S8 lists AIM parameters for the selected bonds. Within structures of derivatives, there are several types of bonds. The first type includes carbon bonds of aromatic rings. These bonds are characterized by an electron density of 0.3 a.u. and Laplacian between −0.84 and −0.89 a.u, making them the strongest coumarin backbone bonds. A single bond between C1 and C2 has a lower electron density value (0.25–0.27 a.u.). The double bond between carbon and oxygen atoms has an electron density of 0.41 a.u. and Laplacian −0.24 a.u. The C−Br bond is the weakest when bonds with substituents are concerned (electron density equal to 0.15 a.u. and Laplacian equal to −0.14 a.u.). Bonds between carbon atoms and OH/OMe are almost equal in strength and do not depend on the group attached to the oxygen atom. These bonds have a higher electron density value than those between a carbon atom and a nitro group (electron density of 0.26 a.u.). These values nicely follow the possibility of the electron density exchange between the aromatic core and substituent. The positive and negative resonant effects between substituents and the aromatic core are essential for stabilizing and distributing electron density. It is also important to observe that the present substituents do not significantly influence the carbon-carbon bonds surrounding position C6. This proves the assumption that the electron density is exchanged between the coumarin core as a whole with the substituents and that the overall stability is preserved. On the other hand, the choice of substituents can influence the possible interactions with the proteins and free radicals, as investigated in the last section.

### 2.2. Experimental and Theoretical NMR Spectra

The experimental NMR spectra of coumarin derivatives were recorded in DMSO as a solvent and shown in Figures S1–S8. The theoretical chemical shifts were calculated for the structures optimized in DMSO at B3LYP-D3BJ/6-311++G(d,p) level of theory. The structure of TMS was optimized at the same level of theory, and calculated chemical shifts are shown relative to chemical shifts of hydrogen and carbon atoms of TMS to mimic the experimental conditions. The experimental and theoretical values of chemical shifts are presented in Table 2 (**6-Br** as an example) and Tables S9–S11. The notation of carbon atoms follows a scheme shown in Figure 2. These sets were compared by calculating the correlation coefficient and MAE values, as previously defined. The calculated ^13^C NMR chemical shift values were systematically overestimated because of the explicit solvent effect, and the correction factor (0.95) was determined from the dependency between experimental and theoretical values.

The ^1^H NMR spectrum of investigated coumarin derivatives is relatively simple due to the rigid structure of the parent molecule that consists of aromatic and pyrone rings. The ^1^H NMR spectrum of **6-Br** contains a singlet at 3.85 ppm assigned to the methyl group of the aliphatic chain. The corresponding peak in the theoretical spectrum is located at 3.88 ppm. The hydrogen atoms attached to aromatic carbon atoms have the following chemical shifts 7.40, 7.90, and 8.20 ppm. The hydrogen atom attached to a carbon atom of the pyrone ring also has a high chemical shift value (8.70 ppm). The theoretical values of ^1^H NMR differ on average for 0.1 ppm with a high correlation coefficient. The values of chemical shifts are higher than expected due to the proximity of oxygen atoms and the sp^2^ hybridization of neighboring carbon atoms. Chemical shifts of hydrogen atoms within the other three coumarin derivatives (Tables S9–S11) depend slightly on the electronic nature of the substituent. In the case of nitro group substituent, the chemical shift values are somewhat higher than in the case of **6-Br** (3.94 vs. 3.85 ppm for methyl group, etc.). The chemical shifts of hydrogen atoms within **6-OH** and **6-OMe** have almost unchanged values when compared to **6-Br**. Due to the oxygen atom’s proximity, the OMe substituent’s hydrogen atoms have chemical shift values equal to 3.76 in the form of a singlet. In all three cases, the correlation coefficients are higher than 0.999, with MAE values between 0.10 and 0.13 ppm.

The ^13^C NMR spectra allow better comparison between experimental and theoretical values because singlets are exclusively observed. The lowest chemical shift value in the ^13^C NMR spectrum of **6-Br** was obtained for the carbon atom of a methyl group (53 ppm in the experimental and 55 ppm in the theoretical spectrum). Carbon atoms in positions 3, 8, and 10 have chemical shifts around 120 ppm, an expected range for the aromatic carbon atoms. Once the electronegative oxygen atoms are present adjacent to carbon atoms, their chemical shifts increase to 132 ppm. Br as a substituent leads to a chemical shift of 137 ppm for the neighboring carbon atom in the experimental and theoretical spectra. The largest chemical shift value was obtained for the carbon atom of the ester group (C1’), namely 163 ppm in the observed spectrum and 167 ppm in the theoretical spectrum. The correlation between measured and calculated values was 0.995, with MAE equal to 2 ppm. The chemical shift of the carbon atom to which the NO_2_ group is linked is 129 ppm (132 ppm in the theoretical spectrum), whereas the chemical shifts of the neighboring carbons C5 and C7 are lowered by several ppm. The rest of the values are almost identical. The same applies to **6-OH** and **6-OMe** coumarin derivatives. The correlation coefficients in these three cases are higher than 0.995, with MAE values of 2 ppm. These results prove that the selected level of theory is suitable for the investigated systems. In the following two sections, the comparison between experimental and theoretical IR and UV-VIS spectra is performed.

### 2.3. Experimental and Theoretical IR Spectra

**Figure 3:**
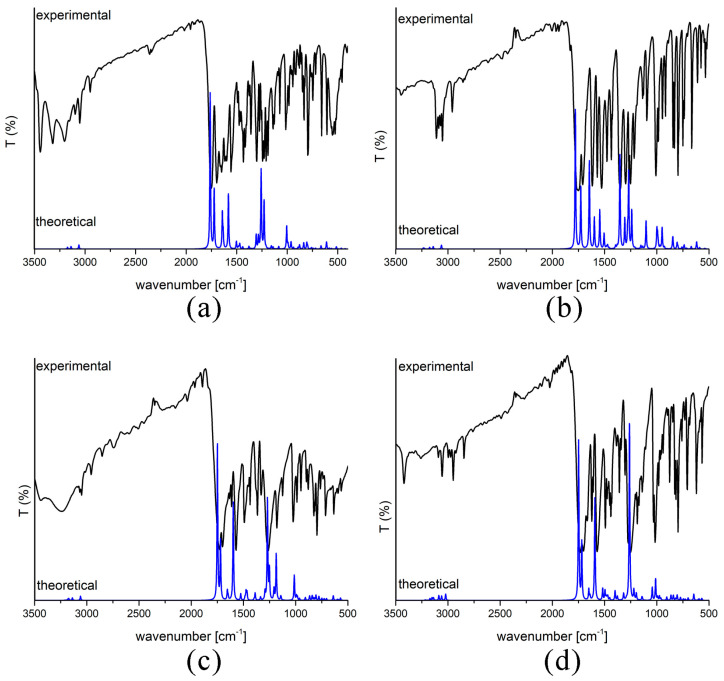
IR spectra of (**a**) **6-Br**, (**b**) **6-NO_2_**, (**c**) **6-OH**, and (**d**) **6-OMe** (experimental—black line, calculated at B3LYP-D3BJ/6-311G(d,p) level of theory—blue peaks).

The IR spectra of four coumarin derivatives were obtained for the compounds in the solid phase within the KBr pallet between 4000 and 400 cm^−1^. The theoretical spectra were calculated for isolated compounds in a vacuum and visualized in the GaussView 6. The theoretical values reproduced the experimental ones well, as observed in the most prominent peaks belonging to C=O stretching vibrations, which was additional proof that the selected level of theory was suitable for this class of compounds. The experimental and theoretical spectra of all derivatives are shown in Figure 3. The analysis of IR spectra is separated into three regions, with the most prominent bands explained.

The first region is between 4000 and 2900 cm^−1^ and includes different X−H (X= C and O) stretching vibrations. In the case of **6-Br**, this region is dominated by the broad peak of C_aromatic_−H stretching vibrations at 3200 cm^−1^ in the experimental and 3138 cm^−1^ in the theoretical spectrum. The weaker band is at 3043 cm^−1^, attributed to the C−H stretching vibrations of a methyl group. This wavenumber is somewhat higher than expected due to the oxygen atom directly attached to the methyl group [28]. The same peaks can be observed for the other three derivatives (Figure 3). A noticeable difference exists in the experimental IR spectrum of **6-OH** with a broad peak assigned to O−H stretching vibration at 3242 cm^−1^. This band is a weak peak at 3400 cm^−1^ in the theoretical spectrum. The bathochromic shift in the experimental spectrum is due to the physical state of the sample and the possible formation of hydrogen bonds between molecules of investigated compounds.

The second part of the spectrum, between 1800 and 1000 cm^−1^, also contains several prominent peaks of different vibrational modes. Two peaks at 1751 and 1698 cm^−1^ in the experimental spectrum of **6-Br** belong to the C=O stretching vibrations of two carbonyl groups. The first peak is assigned to the carbonyl group of the pyrone moiety. These two peaks are located at 1759 and 1723 cm^−1^ in the theoretical spectrum. This difference in values is acceptable for the calculated values bearing in mind that the spectra were predicted for the compounds in a vacuum [28]. The effect of electron-donating/withdrawing substituents can be followed on these bands in the theoretical spectrum. In the case of **6-NO_2_,** there is a hypsochromic shift in the position of these two bands (1781 and 1726 cm^−1^) due to the electron-withdrawing nature of the substituent. A much stronger influence is observed on the position of the C=O group of the pyrone ring that is part of the molecule backbone. The effect on the alkyl chain in position 3 is much weaker. As previously discussed, the electron-donating effect of OH and OMe groups increases the electron density within a molecule and leads to structural relaxation. The excess electron density leads to the bathochromic shift in wavenumber values. The peaks of C=O stretching vibration in the case of **6-OH** (1749 and 1714 cm^−1^) and **6-OMe** (1742 and 1722 cm^−1^) are bathochromically shifted. This region contains C−O stretching vibrations due to the ester group of the substituent in position 3. Spectra of **6-OH** and **6-OMe** have a strong band at 1270 cm^−1^ attributed to the C_aromatic_−O stretching vibration. Due to the system’s rigidity, this value shifts towards larger wavenumbers than C_aliphatic_−O stretching vibration. The nitro group has several characteristic vibrations [33,34]. The symmetric stretching vibration of the nitro group was located at 1340 cm^−1^ in the experimental spectrum and 1349 cm^−1^ in the theoretical spectrum of **6-NO_2_** as strong bands. The asymmetric stretching vibration of the nitro group is positioned at higher wavenumbers (1537 cm^−1^ in experimental and 1541 cm^−1^ in theoretical spectra).

The spectrum between 1000 and 500 cm^−1^ mainly includes medium to low-intensity bands of bending, torsion, and out-of-plane vibrations [12,35]. When different substituents are concerned, this region has several important bands. The C−Br stretching vibration is located at 660 cm^−1^ in the experimental and 665 cm^−1^ in the theoretical spectrum of **6-Br**. At 845 cm^−1^, a deformation vibration of the nitro group can be observed in the spectrum of **6-NO_2_** [33]. The position of this band is shifted towards higher wavenumbers due to the proximity of the aromatic ring. Additionally, there are rocking vibrations of the nitro group at 532 cm^−1^. All of these values are well-reproduced in the experimental spectrum.

### 2.4. Experimental and Theoretical UV-Vis Spectra

**Figure 4:**
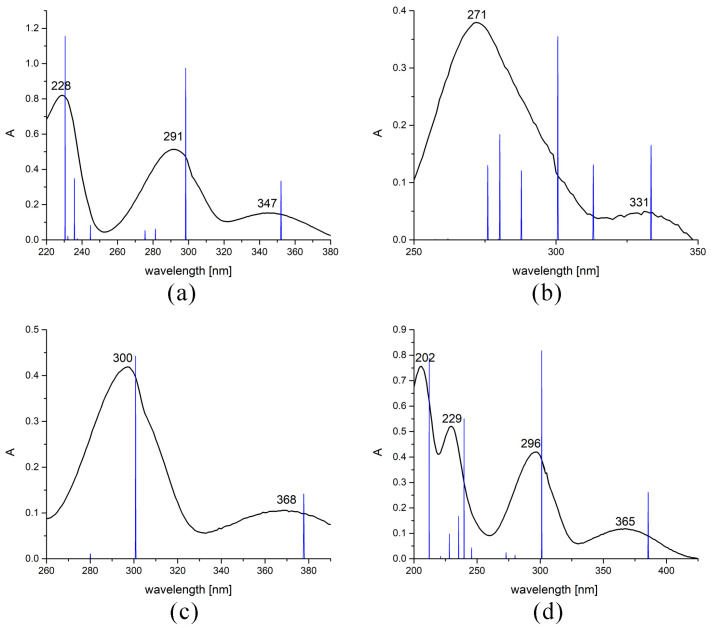
UV-Vis spectra of (**a**) **6-Br**, (**b**) **6-NO_2_**, (**c**) **6-OH**, and (**d**) **6-OMe** (experimental—black line, calculated at B3LYP-D3BJ/6-311G(d,p) level of theory—blue peaks).

The experimental UV-Vis spectra of four derivatives were recorded in ethanol between 800 and 200 nm. The theoretical UV-Vis spectra were calculated for the structures reoptimized in ethanol using the CPCM model. The experimental spectra of derivatives are shown in Figure 4. The electronic spectrum of **6-Br** is characterized by three broad peaks between 220 and 380 nm (347, 291, and 228 nm). The first peak is attributed to n→π transition, while the other two can be assigned as π→π transitions. As previously stated, Br influences the rest of the molecule through a weak resonance effect. The peaks shift towards longer wavelengths once this atom is exchanged with the OH group, as Figure 4 shows, due to the strong positive resonant effect. The first peak shifts to 368 nm, while the second shifts to 300 nm. The methoxy group also donates electron density to the rest of the molecule through a strong positive inductive effect. The shifts are lower than in the case of the OH group. When the NO_2_ group is present in a molecule, the dominant effect is electron withdrawal from the other parts of the molecule, leading to the hypsochromic shift, and the peaks are positioned at 331 and 271 nm. These electronic spectra changes nicely follow the expected electron donating/withdrawing effects of substituents [24]. The addition of various substituents can be important for the synthesis of novel fluorophores [36].

The theoretical spectra of four derivatives were predicted at the B3LYP-D3BJ/6-311++G(d,p) level of theory upon optimization in ethanol to mimic the experimental conditions (Figure 4). The longest wavelength peak in the spectrum of **6-Br** is located at 351 nm. This transition is assigned to the HOMO→LUMO transition (96%) with an oscillator strength of 0.1344 (Table S12). The second and third prominent transitions are at 298 and 230 nm, assigned to HOMO-1→LUMO (85%) and HOMO→LUMO+1 (38%)/HOMO→LUMO+2(45%), respectively. The relative intensity order of the mentioned transitions is the same as for the experimental peaks. These results prove that the theoretical spectrum reproduces the experiment well. The differences of several nm can be explained by the solvent effect and formation of specific solvent–solute interactions, which are not included in the used solvent model. The theoretical assignments for the other three compounds are presented in Table S12. The calculated values of electronic transitions are well correlated with the experimental ones, especially in the case of **6-OH** and **6-OMe**, with differences of several nm. As explained previously, the computed electronic transition values shift predictably, which is in line with the NBO and QTAIM analyses of the intramolecular effects. In the case of **6-NO_2_**, a broad peak at 271 in the experimental spectrum probably presents an overlap between several transitions to energetically similar excited states due to the n orbitals of heteroatoms of the NO_2_ group (Figure 4). The other peak is at 331 nm in the experimental and 333 nm in the theoretical spectrum. This type of analysis again proved the applicability of the chosen level of theory.

### 2.5. EPR Measurements of Antioxidant Activity

**Figure 5:**
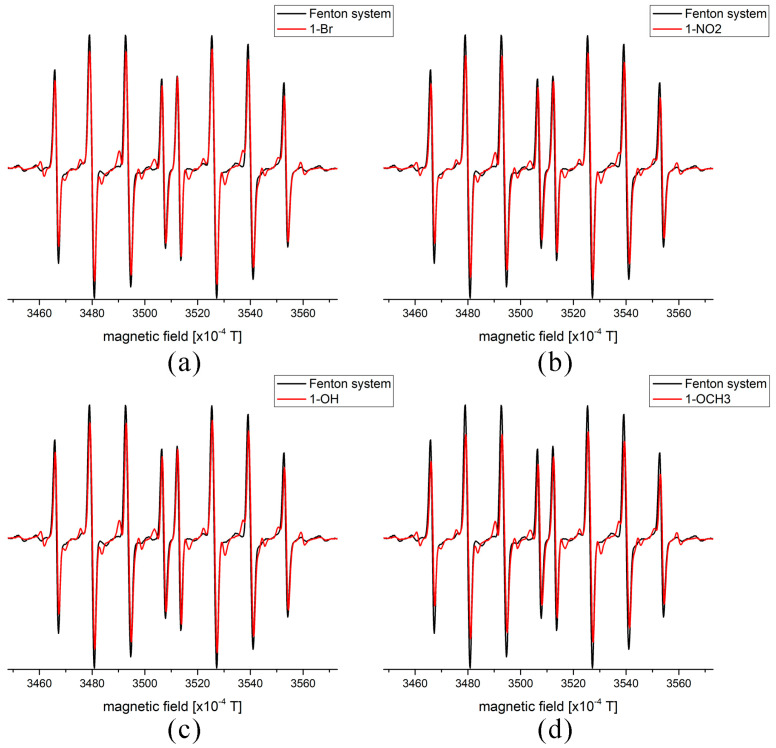
The experimental EPR spectra of DEPMPO/HO^•^ adducts with and without compounds (**a**) **6-Br**, (**b**) **6-NO_2_**, (**c**) **6-OH**, and (**d**) **6-OMe**.

It has been previously shown that coumarin derivatives are effective radical scavengers in biological systems and through advanced oxidation processes in wastewater management [37,38,39]. The reactivity of chosen coumarin derivatives, at 10^−5^ M final concentration, towards HO^•^ was followed by the EPR spectroscopy through a relative decrease in the DEPMPO/HO^•^ signal. The EPR spectra before and after adding coumarin derivatives are shown in Figure 5 below.

Figure 5 shows that the signal of DEPMPO/HO^•^ decreases upon the addition of investigated coumarin derivatives, proving their anti-radical activity. The radical scavenging activities calculated as reduction percentages are 23% (**6-OMe**), 16% (**6-OH**), 15% (**6-NO_2_**), and 13% (**6-Br**). The similarity in these values was expected as selected derivatives do not contain groups usually responsible for anti-radical activity, except in the case of **6-OH**. Based on these values, the investigated compounds can be considered moderate scavengers compared to standard antioxidants such as fisetin, baicalein, quercetin, morin, and kaempferol, which reduce the signal of the DEPMPO/HO^•^ adduct between 30 and 43% [40]. The activity of these compounds was lower than that of 4-hydroxycoumarin under the same experimental conditions [38]. The relative order of anti-radical activity can be explained by the electron-donating/withdrawing effects of substituents. Two coumarin derivatives with substituents characterized by the strongest electron donation have higher scavenging activity values, thus proving the importance of electron delocalization for activity. The possible mechanism of activity can be postulated to radical adduct formation, as there are no substituents present in a structure that commonly donate hydrogen atoms/protons to free radicals [38]. Once the groups with dominant negative inductive and negative resonant effects are present, the aromatic part of the molecule is more destabilized by radical adduct formation. These findings align with the previous discussion on the impact of electron effect on spectra and reactivity.

### 2.6. Spectrofluorometric and Molecular Docking Investigation of Binding to BSA

**Figure 6:**
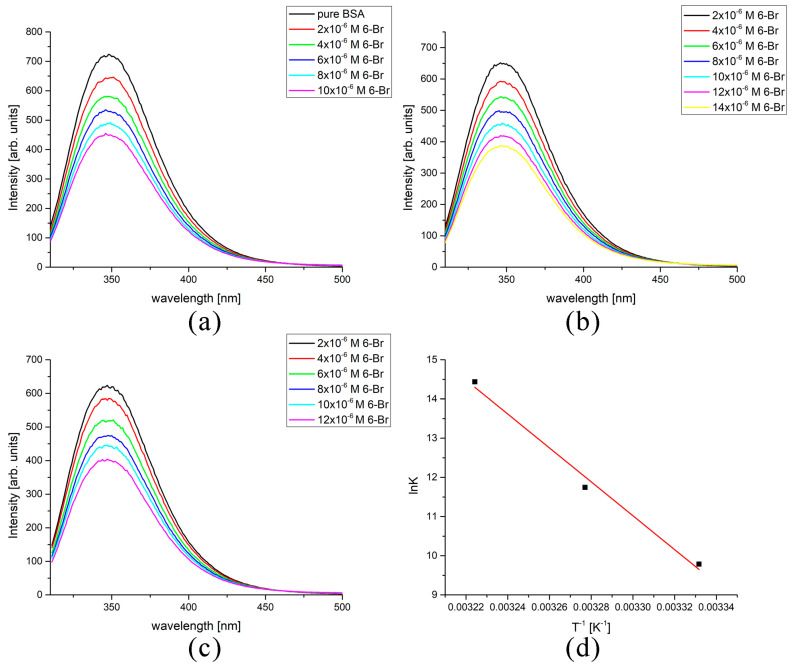
The BSA-binding fluorescence curves for **1-Br** at (**a**) 27 °C, (**b**) 32 °C, and (**c**) 37 °C, and (**d**) Van’t Hoff’s plot for binding.

The binding of compounds to BSA influences the possibility of their distribution throughout the organism. In this contribution, the binding process was investigated experimentally and through molecular docking simulations to examine the effect of substituents on the binding energy and interactions with surrounding amino acids in the active pocket. The spectra of BSA before and after the addition of compounds at three different temperatures are provided in and Figure 6 and Figures S9–S11. The double-log Stern–Volmer relationship was used to calculate binding constants and obtain the thermodynamic parameters of binding through their change with temperature. A temperature range between 27 and 37 °C was selected to cover normal body temperature so the conclusion on interactions under physiological conditions can be obtained. The paper shows the spectra of **6-Br** binding to BSA as a representative example. Table 3 lists all four coumarin derivatives’ binding constants and thermodynamic parameters.

As observed in and Figure 6 and Figures S9–S11, fluorescence emission intensity decreased concentration-dependently upon adding coumarin derivatives. The correlation coefficients for the double-log Stern–Volmer plots are provided in Table 3; for all data sets, they are between 0.97 and 0.99. The decrease in fluorescence intensity is similar for all compounds except for **6-OMe**. In this case, an isosbestic point can be seen in Figure S11, in addition to the appearance of a new peak at 480 nm. The explanation for this peak is probably due to the formation of covalent bonds between coumarin derivatives and amino acids surrounding the active positions, as **6-OMe** itself is not fluorescent. These changes in spectra were not further investigated, although it would be essential to understand the possible underlying mechanism.

The dependence of lnK to reciprocal temperature is linear for all compounds, which allowed the determination of the thermodynamic parameters of binding. In the case of **6-Br** and **6-NO_2_,** the dependence has a negative slope, leading to the positive value of ΔH_bind_, 556 and 360 kJ mol^−1^, respectively. These values are −254 (**6-OH**) and −236 kJ mol^−1^ (**6-OMe**) for the other two derivatives. According to data shown in Table 3, the most spontaneous binding at 37 °C was between BSA and **6-NO_2_** (−41.0 kJ mol^−1^), followed by **6-Br** (−36.7 kJ mol^−1^). Somewhat lower ΔG_bind_ values were calculated for **6-OH** (−24.6 kJ mol^−1^) and **6-OMe** (−23.7 kJ mol^−1^). The reason probably lies in the formed interactions with the surrounding amino acids in the active pocket, as investigated further. With the decrease in temperature, between 37 and 27 °C, the ΔG_bind_ values decrease in the case of **6-NO_2_** and **6-Br** and increase for the other two derivatives. This leads to the conclusion that the dominant factor for binding is the change in reaction entropy. With the decrease in temperature, the movement of routable substituents such as OH and OMe decreases, allowing the formation of stronger interactions, which proves the importance of the substituent’s flexibility for the binding process. Further experimental techniques for analyzing the changes in BSA confirmation include circular dichroism, gel electrophoresis, and viscosity measurements.

Molecular docking analysis was performed on human serum albumin (HSA), a protein with similar active pockets as BSA. HSA is more relevant for biological studies and was therefore selected for molecular docking analysis. As it is well known in the literature, HSA has several binding sites, with the main two being named “Sudlow I” and “Sudlow II,” also named as “warfarin binding site” and “benzodiazepine/ibuprofen binding site”, respectively. Sudlow site I is under subdomain IIA, whereas Sudlow site II is in subdomain IIIA. Sudlow site I preferentially bind large heterocyclic chemicals such as azapropazone, phenylbutazone, and warfarin (WF). Sudlow site II predominantly binds aromatic chemicals such as ibuprofen (IP). The binding affinities for the most stable conformations in both active pockets for all four coumarin derivatives are presented in Table 4. Warfarin and ibuprofen were docked into Sudlow I and II active pockets, along with investigated compounds.

According to the results of molecular docking simulations presented in Table 4, the highest binding affinity was exhibited by compound **6-NO_2_** (−31.4 kJ mol^−1^ in Sudlow 1 and −30.9 kJ mol^−1^ in Sudlow II), followed closely by the compounds **6-Br**, **6-OH**, and **6-OMe**. The calculated range of values and the relative affinity order nicely follow the experimentally determined one. This also verifies the assumption that the molecular docking study with HSA can model the binding process to BSA. Interestingly, compounds with bromine and methoxy groups have slightly higher binding affinity at Sudlow II than the first active pocket (−30.1 vs. −28.5 kJ mol^−1^ for **6-Br**, for example). In the case of the compound with a nitro group, higher affinity was calculated at Sudlow I. Compound **6-OH** shows equal binding affinity towards both sites. The binding affinity of warfarin at Sudlow I is the highest among the investigated compounds (−34.3 kJ mol^−1^). The binding of Ibuprofen at Sudlow II is thermodynamically less favorable (−28.3 kJ mol^−1^) compared to **6-Br**, **6-NO_2_**, and **6-OH**.

**Figure 7:**
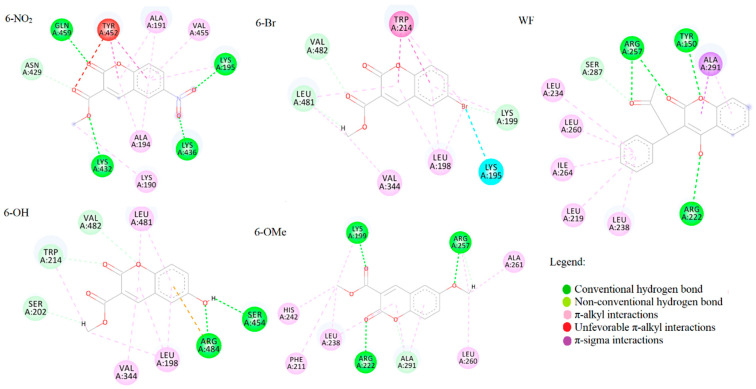
Interactions of WF and four coumarin derivatives with HSA at Sudlow I binding site.

Binding energies from Table 4 indicate lower binding affinity of investigated compounds than WF, which can be explained by closer examination of protein–ligand interactions at the active site. As can be seen from Figure 7, the strongest interactions WF makes are four conventional hydrogen bonds with ARG257, TYR150, and ARG222. These amino acid residues represent the active site Sudlow I.

**Figure 8:**
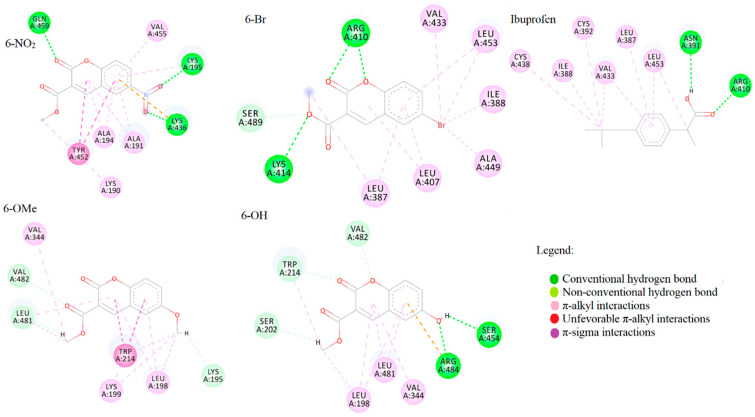
Interactions of IP and four coumarin derivatives with HSA at Sudlow I binding site.

Data from Figure 8 suggest that 6-OMe forms hydrogen bonds with two of the three mentioned amino acid residues due to the highest similarity to ibuprofen. Additionally, **6-OMe** forms a hydrogen bond with LYS199. Regarding π–π interactions, LEU230 and LEU238 are amino acids that interact with WF and **6-OMe**. The highest number of conventional hydrogen bonds, alongside the WF, is built between the **6-NO_2_** and GLN459, LYS195, LYS432, and LYS432, respectively. However, **6-NO_2_** with TYR452 forms an unfavorable π–π interaction, reflected in binding energies higher than WF’s, despite the same number of conventional hydrogen bonds. The number of conventional hydrogen bonds at Sudlow site II directly correlated to the binding energies and investigated compounds’ overall binding potential. The compound with a nitro group formed three conventional hydrogen bonds with GLN459, LYS195, and LYS 436, respectively (Figure 8), consequently showing the lowest binding energies. It was followed closely by **6-Br**, forming three conventional hydrogen bonds, two with ARG410 and one with LYS414. Ibuprofen and **6-OH** formed two conventional hydrogen bonds, while **6-OMe** showed no conventional hydrogen bonds.

If further examined, data from Table 4 indicate the binding potential of the investigated compounds similar to the binding potential of IP in Sudlow site II. These structures will be further examined by molecular dynamics simulations. However, the overall binding affinity of all investigated compounds was good, indicating the high possibility of transport of investigated compounds by albumin throughout the organism.

### 2.7. Molecular Dynamics

**Figure 9:**
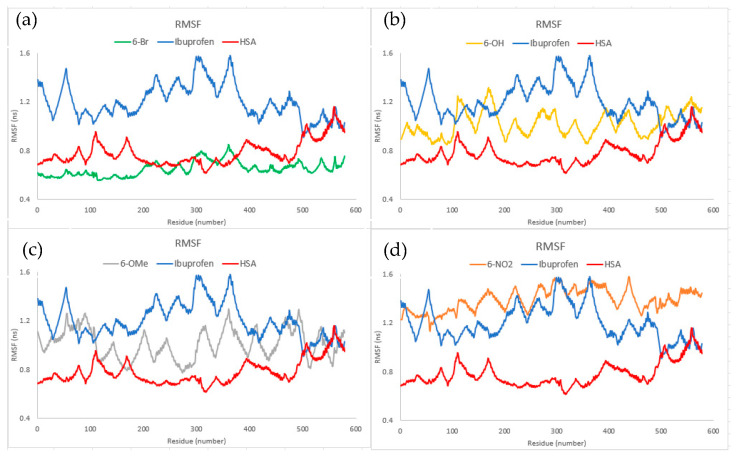
RMSF diagrams describing interactions of (**a**) **6-Br**, (**b**) **6-OH**, (**c**) **6-OMe**, and (**d**) **6-NO_2_** with HSA compared to ibuprofen.

**Figure 10:**
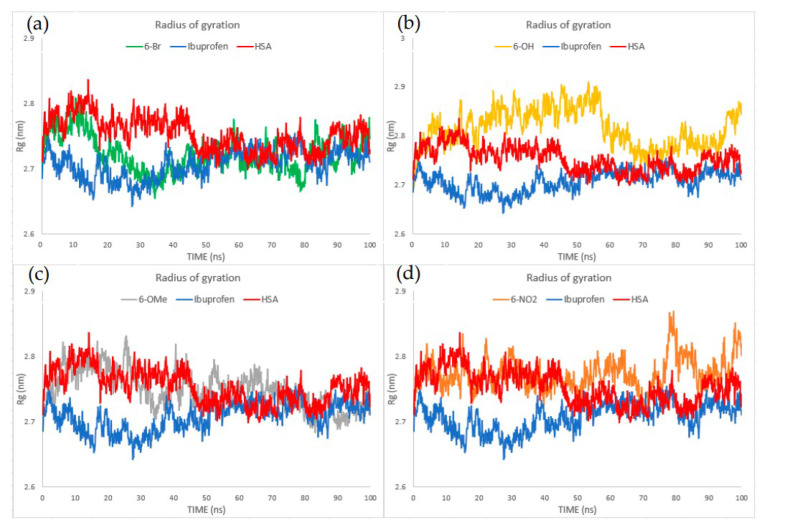
Rg diagrams describing interactions of (**a**) **6-Br**, (**b**) **6-OH**, (**c**) **6-OMe**, and (**d**) **6-NO_2_** with HSA compared to ibuprofen.

Molecular dynamics simulations were performed to investigate further results obtained through molecular docking. According to the RMSD values in Figure S12, the binding of investigated compounds has a comparable effect on changes in RMSD values as ibuprofen. This indicates that the chemical behavior of the investigated compounds, in terms of altering the secondary structure of HSA, is comparable to that of ibuprofen. Additionally, RMSD values of HSA itself show no significant changes in the secondary structure, regardless of the bonded ligand. However, larger differences were observed when examining the RMSF and Rg values (Figure 9 and Figure 10). According to the RMSF values, the stabilizing effect of binding **6-OH** and **6-OMe** on the flexibility of HSA amino acid residues was comparable but slightly higher than that of ibuprofen. An even greater stabilization of amino acid residues was observed when HSA was complexed with **6-Br**. In contrast, the binding of **6-NO_2_** increased the flexibility of HSA amino acid residues compared to the binding of ibuprofen.

Regarding the flexibility of amino acid residues in HSA in the absence of ligands bound to its active sites, it was observed that the binding of all investigated compounds, including ibuprofen, led to an increase in residue flexibility, except for **6-Br**. When it comes to changes in the compactness of HSA, the binding of **6-Br** and **6-OMe** has a similar effect as the binding of ibuprofen. On the other hand, the binding of **6-NO_2_**, especially **6-OH**, reflects the increase in the radius of gyration, indicating changes in the compactness of the HSA in comparison to the HSA–ibuprofen complex. Concerning the HSA itself, the binding of ibuprofen and **6-Br** increases protein compactness, whereas the binding of **6-OMe** and **6-NO_2_** does not induce any significant changes in protein compactness. The Rg values are in reverse correlation with the binding energies and inhibitory constants obtained through molecular docking simulations. However, RMSF values cannot be correlated with binding energies, but they can be correlated to the charge distribution throughout the molecule, which has, as a consequence, different types and numbers of interactions with HSA amino acid residues. For example, Br is a substituent with a positive resonant and negative inductive effect, and because the negative charge is fairly localized on the bromine atom, it forms several electrostatic interactions with amino acid residues (Figure 6). This minimizes the flexibility of amino acid residues locally with lower RMSF values. The formation of strong hydrogen bonds between amino acids in the active pocket and OH, OMe, and NO_2_ groups leads to a significant change in the secondary structure and fluctuations in the positions of atoms, which results in a higher average deviation of the positions.

The presented conclusions are important for biomedical applications as the defined substituent’s effect allows fine-tuning of the activity through synthesizing compounds with desirable moieties. Small differences in electronic effect can lead to differences in interactions with proteins and other biomolecules and the scavenging of free radicals.

## 3. Materials and Methods

### 3.1. Chemicals

The investigated coumarin derivatives were obtained as previously described [41]. Solvents used for the other experiments were purchased from Merck (Merck & Co., Rahway, NJ, USA) as p.a. chemicals.

### 3.2. Spectroscopic Analysis

The IR spectra of investigated coumarin derivatives were recorded on a Thermo Nicolet-Avatar 370 FTIR spectrometer (Thermo Fisher, Waltham, MA, USA) in the range between 4000 and 400 cm^−1^. The samples were prepared in the KBr pallet with the mass ratio of derivatives: KBr = 2 mg: 150 mg. The UV-VIS spectra were obtained in ethanol on the Thermo Scientific–Evolution 220 spectrophotometer (Thermo Fisher, Waltham, MA, USA) between 800 and 200 nm. The NMR spectra were measured on a Bruker AvanceTM 400 MHz spectrometer (Bruker, Billerica, MA, USA). Chemical shifts are shown relative to TMS.

### 3.3. Spectrofluorimetric Analysis of BSA Protein Binding

The affinity of coumarin derivatives towards bovine serum albumin (BSA) was investigated by the spectrofluorimetric measurements on a Cary Eclipse MY2048CG03 instrument (Agilent Technologies, Santa Clara, CA, USA). The excitation wavelength was set to 295 nm, corresponding to the excitation of tryptophan residues, and emission was followed between 310 and 500 nm. The scan rate was 600 nm min^−1^, with both slits set to 5 nm. The concentration of BSA in phosphate-buffered saline was kept constant at 5 × 10^−5^ M, while the concentration of coumarin derivatives changed between 0.1 and 1 × 10^−5^ M. The measurements were performed at three temperatures (27, 32, and 37 °C) to allow the calculation of the thermodynamic parameters that govern the binding process. The decrease in fluorescence emission intensity followed a double logarithmical Stern–Volmer quenching mechanism.

### 3.4. Electron Paramagnetic Resonance Spectroscopy (EPR) Analysis of Radical Scavenging Activity

The anti-HO^•^ activity of compounds was measured by the electron paramagnetic resonance (EPR) spectroscopy on a Bruker Elexsys E540 EPR spectrometer (Bruker, Billerica, MA, USA) operating at X-band (9.51 GHz). The following parameters for the measurements were set: modulation amplitude—1G; modulation frequency—100 kHz; microwave power—10 mW, center field—3500 G. The spectra were recorded using Xepr software 3 (Bruker BioSpin, Billerica, MA, USA). The samples were drawn into 5 cm long gas-permeable Teflon tubes (Zeus Industries, Raritan, Franklin Township, NJ, USA) with a wall thickness of 0.025 mm and an internal diameter of 0.6 mm. The measurements were performed under normal conditions, using quartz capillaries into which Teflon tubes were placed. The radical was obtained in the Fenton system with the following concentrations: 5 mM H_2_O_2_, 5 mM FeSO_4_, and 100 mM spin-trap DEPMPO. The amount of radical was determined by the EPR signal after the formation of spin-adduct with DEPMPO. Due to the compound’s insolubility in water, a 10 mM solution of coumarin derivatives was prepared in DMSO and diluted with water to 10^−4^ M. The final concentration of coumarin derivatives in each measurement was 10^−5^ M. The blank probe contained only the Fenton system with the same amount of DMSO as the other measurements. The radical scavenging activity of compounds was calculated from the peak heights as the relative decrease of the EPR signal of spin-adduct before and after the addition of compounds. The activity was calculated as the *% of reduction* = 100 × (I_0_ − I_a_)/I_0_. The intensities in the previous equation denote the intensities of the second and third low-field EPR peaks of the control system and a sample containing coumarin derivatives, respectively.

### 3.5. Theoretical Methods

Structure optimizations were performed in the Gaussian 09 Program package Version 09 [42]. Three common functionals (B3LYP-D3BJ, APFD, and M06-2X) [43,44,45,46] in conjunction with the 6-311++G(d,p) [47] basis set were employed for the optimization of the structure. These calculations were conducted without any geometrical constraints. The absence of imaginary frequencies proved that the minima on the energy surface were obtained. The vibrational modes were visualized in the GaussView 6 program [48] and further investigated through the potential energy distribution (PED) analysis. The conductor-like polarizable continuum (CPCM) solvent model was employed to mimic the experimental conditions [49]. The electronic transitions were calculated by the time dependent-density functional theory (TD-DFT) in ethanol as the solvent. The ^1^H and ^13^C NMR spectra of differently substituted coumarin derivatives were obtained by the gauge independent orbital approach (GIAO) [50,51], which was implemented in the Gaussian09 Program package [42]. The calculated values of chemical shifts are presented relative to the signals of TMS, optimized at the same level of theory. The effects of substituents were analyzed in detail by the natural bond orbital (NBO) and quantum theory of atoms in molecules (QTAIM) methods. NBO [52] is used to access the energy of stabilization interactions that govern structure stability. On the other side, QTAIM is a complementary approach to investigate the intermolecular interactions based on the electron density and its Laplacian in the bond critical points (BCP) and ring critical points (RCP) [53]. This approach is based on Bader’s theory of interacting atoms. These calculations were carried out in the AIMAll program package [54]. QTAIM recognizes two types of interactions: closed and open-shell interactions [53,55]. The first type includes covalent bonds with an electron density of around 0.1 a.u. and large negative Laplacian. The second type covers ionic bonds, van der Waals interactions, and hydrogen bonds. The electron density of these interactions is between 0.001 and 0.04 a.u., while Laplacian has a positive value.

### 3.6. Molecular Docking Analysis

Molecular docking studies were performed to investigate the binding affinity towards transport proteins and evaluate binding energies and inhibitory constants. The crystal structure of the protein used in the molecular docking study (human serum albumin (HSA)) was obtained from the RCSB Protein Data Bank with PDB ID:2BXD [56]. Water molecules, cofactors, and co-crystalized ligands were deleted, and protein was prepared for the simulation using BIOVIA Discovery Studio 4.0. [57] As previously mentioned, ligands were prepared for simulations by geometry optimization using the Gaussian09 software package. For performing molecular docking simulations, the AMDock software package with implemented AutoDock 4.2.6 was used [58]. The Kollman partial charges and polar hydrogens were added using the AutoDockTools interface. The flexibility of the ligands/complexes was considered during simulations, while the protein remained rigid. The Lamarckian genetic algorithm (LGA) was employed for protein-complex flexible docking. The following parameters were determined for the LGA method: there were a maximum of 250,000 energy evaluations, 27,000 generations, and mutation and crossover rates of 0.02 and 0.8, respectively. For the search of the active site and ligand orientation, AutoGridFR was utilized. AutoDock 4.2.6 was implemented for the molecular docking energy calculations using Amber Force Field [59,60,61]. The interactions between the target protein and the investigated compounds were analyzed and illustrated in 3D using BIOVIA Discovery Studio 4.0 and ADT.

### 3.7. Molecular Dynamics Analysis

Structures from the molecular docking which express the highest binding potential were subjected to molecular dynamics simulations (MD). The AMBER22 software package with implemented CHARM36m force field was used to perform MDs [62]. The Charmm-GUI server produced the investigated compounds’ topologies, input parameters, and coordinate files [63]. The steepest descent and conjugate gradient algorithms were used to conduct minimization with a tolerance of up to 1000 kJ mol^−1^ nm^−1^ over 50,000 steps. The phase of equilibration was conducted under NVT ensemble settings. The MD production process was carried out in an NPT ensemble utilizing the SHAKE algorithm for a 100 ns time scale and implementing a Monte-Carlo barostat (P = ps). Additionally, from MD output trajectories, root mean square deviation (RMSD), radius of gyration (Rg), and root mean square fluctuation (RMSF) were calculated to examine system features during and after molecular dynamics simulations, including general stability and structural fluctuations. These parameters are used to assess the stability and structural changes of the protein–ligand complex across the calculated timeframe [64,65].

## 4. Conclusions

The effects of electron-donating/electron-withdrawing substituents on spectral, structural, antioxidant, and protein binding activities were analyzed for four coumarin derivatives. The appropriate DFT level of theory (B3LYP-D3BJ/6-311++G(d,p)) was selected upon the comparison between experimental and theoretical bond lengths and angles of coumarin-3-carboxylic acid and 3-acetylcoumarin as structurally similar compounds. The comparison between structural parameters of four coumarin derivatives showed a resemblance between these structures, with the only difference being the bond length between a carbon atom and a substituent. The main difference in NBO stabilization interactions came from the interaction between lone pair of atoms in substituent (oxygen, nitrogen, and bromine) and surrounding carbon–carbon bonds. These values were nicely followed by QTAIM parameters. The experimental NMR chemical shifts were compared to the calculated ones. The 1H NMR chemical shifts’ correlation coefficient was 0.999 in all cases, with MAE values between 0.1 and 0.13 ppm. Regarding ^13^C NMR chemical shifts, high correlation coefficients and low MAE values were also obtained. The most prominent IR bands were well reproduced, especially for the substituents. UV-VIS spectra were assigned based on theoretical values, with the difference in several nanometers attributed to the explicit solvent effect that was not included in the used solvent model. The percentage of reduction in HO^•^ also depended on the present substituent (23% (**6-OMe**), 16% (**6-OH**), 15% (**6-NO_2_**), and 13% (**6-Br**)), marking the investigated compounds as moderate radical scavengers. All coumarin derivatives interacted spontaneously with BSA, as shown by spectrofluorometry. The binding affinity decreased at body temperature in order **6-NO_2_** > **6-Br** > **6-OH** > **6-OMe**. This order was reproduced by molecular docking simulations. All compounds bind more tightly to HSA than ibuprofen, except for **6-OMe**. The binding affinity of warfarin was higher than for the investigated compounds. The effect of substituent was also shown in MD simulations on the compactness and flexibility of the protein. These results significantly added to the understanding of the electron effect of substituents on biological activities, and further studies are needed to include other typical substituents.

## Figures and Tables

**Table 1:** R and MAE values for comparing experimental and theoretical (with various functionals and 6-311++G(d,p) basis set) bond lengths and angles of **1** and **2**.

		1			2		
		APFD	B3LYP-D3BJ	M06-2X	APFD	B3LYP-D3BJ	M06-2X
Bond lengths	R	0.982	0.981	0.981	0.995	0.996	0.998
	MAE [Å]	0.014	0.015	0.014	0.007	0.006	0.007
Bond angles	R	0.945	0.943	0.936	0.982	0.983	0.979
	MAE [^o^]	1.05	1.01	1.15	0.46	0.43	0.48

**Table 2:** Experimental and theoretical (at B3LYP-D3BJ/6-311++G(d,p)) ^1^H and ^13^C chemical shifts of **6-Br**.

^1^H Chemical Shifts [ppm]			^13^C Chemical Shifts [ppm]		
H Atom	Experimental	Theoretical	C Atom	Experimental	Theoretical
C2′-H	3.85	3.88	C2’	53	55
C8-H	7.40	7.46	C3	117	118
C7-H	7.90	7.84	C8	119	119
C5-H	8.20	7.95	C10	120	121
C4-H	8.70	8.82	C5	132	133
R		0.999	C6	137	137
MAE [ppm]		0.10	C7	148	140
			C4	154	152
			C2	156	157
			C9	160	157
			C1’	163	167
			R		0.995
			MAE [ppm]		2.0

**Table 3:** Thermodynamic parameters of BSA binding.

Derivative	T [K]	lnK	R	ΔH [kJ mol^−1^]	ΔS [kJ mol^−1^ K^−1^]	ΔG [kJ mol^−1^]
**6-Br**	300	9.78	0.98	360	1278	−22.9
	305	11.74	0.98			−30.3
	310	14.44	0.99			−36.7
**6-NO_2_**	300	9.03	0.99	556	1924	−21.8
	305	12.16	0.99			−31.4
	310	16.21	0.99			−41.0
**6-OH**	300	5.39	0.99	−254	−740	−32.0
	305	4.84	0.98			−28.3
	310	3.96	0.99			−24.6
**6-OMe**	300	12.62	0.99	−236	−685	−30.5
	305	9.95	0.98			−27.1
	310	9.58	0.98			−23.7

**Table 4:** Binding energies (in kJ mol^−1^) and inhibitory constants (in nM), which describe the binding of investigated compounds to HSA in Sudlow I and II.

Compound	Sudlow I		Sudlow II	
	ΔG_bind_	K_i_	ΔG_bind_	K_i_
**6-Br**	−28.5	9.87	−30.1	5.18
**6-NO_2_**	−31.4	3.17	−30.9	3.85
**6-OH**	−28.8	9.06	−28.7	9.19
**6-OMe**	−26.3	24.27	−28.1	11.59
Warfarin/*Ibuprofen*	−34.3	0.95	*−28.3*	*10.90*

## Data Availability

Not applicable.

## Supplementary Materials

The supporting information can be downloaded at: https://www.mdpi.com/article/10.3390/ijms241411820/s1.
