# Supplementary material for: The Electronic Effects of 3-Methoxycarbonylcoumarin Substituents on Spectral, Antioxidant, and Protein Binding Properties
Source: Int J Mol Sci. 2023 Jul 23;24(14):11820. doi: 10.3390/ijms241411820 (PMC10380446; doi:10.3390/ijms241411820)
Supplement: Supplementary file 1 [file ijms-24-11820-s001.zip › ijms-2477778-supplementary.pdf]

Supplementary information for:

The Electronic Effects of 3-Methoxycarbonylcoumarin Substituents on Spectral, Antioxidant, and Protein Binding Properties

Jelena Vasić [1], Dušan Dimić [1,*], Marko Antonijević [2], Edina H. Avdović [2], Dejan Milenković [2], Đura Nakarada [1], Jasmina Dimitrić Marković [1], Maja Molnar [3], Melita Lončarić [3], Drago Bešlo [4,*] and Zoran Marković [2]

1 Faculty of Physical Chemistry, University of Belgrade, Studentski trg 12-16, 11000 Belgrade, Serbia
2 Institute for Information Technologies, Kragujevac, University of Kragujevac, Jovana Cvijica bb, 34000 Kragujevac, Serbia
3 Department of Applied Chemistry and Ecology, Faculty of Food Technology Osijek, Josip Juraj Strossmayer University of Osijek, Franje Kuhača 18, 31000 Osijek, Croatia; melita2608@gmail.com (M.L.)
4 Department of Agroecology and Environmental Protection, Faculty of Agrobiotechnical Sciences Osijek, Josip Juraj Strossmayer University of Osijek, Vladimir Prelog 1, 31000 Osijek, Croatia
* Correspondence: dbeslo@fazos.hr (D.B.); ddimic@ffh.bg.ac.rs (D.D.)

Table S1. Experimental and theoretical (various functionals with 6-311++G(d,p) level of theory) bond lengths of coumarin-3-carboxylic acid

3-carboxylic acid coumarin	experimental	APFD	B3LYP-D3BJ	M06-2X
O1-C	1.368	1.351	1.358	1.353
O1-C12	1.375	1.385	1.390	1.376
C6-C8	1.476	1.484	1.487	1.492
C8-O5	1.216	1.200	1.202	1.194
C8-O2	1.315	1.350	1.358	1.347
C6-C10	1.332	1.346	1.347	1.340
C10-C9	1.442	1.436	1.439	1.446
C9-C7	1.381	1.407	1.410	1.402
C7-C12	1.458	1.471	1.472	1.478
C9-C15	1.399	1.402	1.405	1.401
C15-C19	1.377	1.384	1.386	1.384
C19-C17	1.379	1.400	1.401	1.399
C17-C13	1.371	1.385	1.386	1.384
C13-C7	1.406	1.395	1.398	1.395
C12-O4	1.211	1.198	1.200	1.193
R		0.982	0.981	0.981
MAE [Å]		0.014	0.015	0.014

Table S2. Experimental and theoretical (various functionals with 6-311++G(d,p) level of theory) bond lengths of 3-acetylcoumarin

3-acetylcoumarin	Experimental	APFD	B3LYP-D3BJ	M06-2X
O1-C7	1.385	1.393	1.400	1.380
C7-O3	1.211	1.200	1.202	1.196
C7-C8	1.472	1.466	1.466	1.472
C8-C10	1.510	1.504	1.510	1.512
C8-C9	1.360	1.359	1.361	1.352
C10-O5	1.224	1.214	1.218	1.209
C10-C14	1.505	1.500	1.505	1.503
C9-C4	1.434	1.424	1.426	1.434
C4-C6	1.403	1.404	1.405	1.398
C6-O1	1.380	1.351	1.358	1.355
C4-C13	1.411	1.405	1.408	1.404
C13-C12	1.389	1.381	1.382	1.381
C12-C11	1.403	1.401	1.403	1.401
C11-C2	1.391	1.386	1.388	1.384
C2-C6	1.390	1.391	1.393	1.392
R		0.996	0.996	0.998

MAE [Å]	0.007	0.006	0.007

Table S3. Experimental and theoretical (various functionals with 6-311++G(d,p) level of theory) bond angles of coumarin-3-carboxylic acid coumarin

3-carboxylic acid coumarin	Experimental	APFD	B3LYP-D3BJ	M06-2X
O1-C6-C8	110.42	111.72	112.02	111.71
O1-C6-C10	122.91	123.50	123.17	123.87
C6-C8-O5	121.97	124.54	125.08	124.73
C6-C8-O2	113.37	111.45	111.47	111.42
C10-C6-C8	126.66	124.78	124.81	124.43
C6-C10-C9	119.74	119.31	119.54	119.05
C10-C9-C7	118.08	118.22	118.23	118.04
C10-C9-C15	122.23	122.75	122.73	122.85
C9-C15-C19	120.25	120.05	120.14	119.97
C9-C7-C13	120.14	120.59	120.39	120.69
C9-C7-C12	120.78	120.51	120.60	120.62
C15-C19-C17	119.30	120.55	120.50	120.56
C19-C17-C13	122.01	120.06	120.08	120.04
C17-C13-C7	118.61	119.72	119.85	119.62
C13-C7-C12	119.08	118.89	119.01	118.70
C7-C12-O1	117.16	116.21	116.08	116.22
C7-C12-O4	126.66	125.56	125.97	125.38
O4-C12-O1	116.18	118.23	117.95	118.41
R		0.945	0.943	0.936
MAE [º]		1.05	1.00	1.14

Table S4. Experimental and theoretical (various functionals with 6-311++G(d,p) level of theory) bond angles of 3-acetylcoumarin

3-acetylcoumarin	Experimental	APFD	B3LYP-D3BJ	M06-2X
O1-C7-O3	115.63	116.64	116.44	117.07
C1-C7-C8	116.60	116.12	115.95	116.17
O3-C7-C8	127.77	127.24	127.61	126.77
C7-C8-C9	119.92	119.80	119.88	119.92
C7-C8-C10	121.77	122.53	122.43	122.17
C8-C10-O5	118.79	118.57	118.58	118.37
C8-C10-C14	119.92	120.10	120.18	120.10
O5-C10-C14	121.29	121.33	121.25	121.53

C8-C9-C4	121.94	122.22	122.32	121.90
C9-C4-C6	117.51	117.17	117.33	117.20
C9-C4-C13	123.76	123.93	123.91	123.91
C4-C6-O1	120.80	121.16	120.83	121.09
C4-C6-C2	122.23	121.19	121.28	121.34
C4-C13-C12	119.69	120.27	120.31	120.26
C13-C12-C11	120.03	119.77	119.81	119.76
C12-C11-C2	121.38	121.12	121.05	121.05
C11-C2-C6	117.94	118.76	118.78	118.69
C2-C6-O1	116.97	117.65	117.89	117.58
C6-O1-C7	123.00	123.52	123.70	123.73
R		0.982	0.983	0.979
MAE [º]		0.46	0.43	0.48

Table S5. Optimized bond lengths (in Å) of coumarin derivatives (at B3LYP-D3BJ/6-311++G(d,p) level of theory)

	**6-Br**	**6-NO2**	**6-OH**	**6-OCH3**
C3-C4	1.360	1.358	1.362	1.363
C3-C2	1.465	1.464	1.464	1.463
C3-C1’	1.493	1.495	1.492	1.492
C1’=O	1.215	1.214	1.216	1.216
C1’-O	1.332	1.330	1.333	1.333
O-C2’	1.446	1.446	1.445	1.445
C2=O	1.206	1.202	1.208	1.208
C2-O	1.392	1.401	1.386	1.388
O-C9	1.361	1.352	1.366	1.365
C9-C10	1.402	1.406	1.401	1.399
C9-C8	1.392	1.395	1.392	1.394
C10-C4	1.427	1.429	1.426	1.425
C7-C8	1.387	1.382	1.385	1.382
C7-C6	1.401	1.401	1.406	1.411
C6-C5	1.380	1.382	1.384	1.386
C5-C10	1.407	1.400	1.408	1.411
C6-Br	1.915			
C6-N		1.466		
N-O		1.227		
N-O		1.227		
C6-O			1.365	
C6-O				1.359
O-Me				1.430
MAE	/	0.003	0.002	0.003

Table S6. Optimized bond angles (in °) of coumarin derivatives (at B3LYP-D3BJ/6-311++G(d,p) level of theory)

	**6-Br**	**6-NO2**	**6-OH**	**6-OCH3**
C4-C3-C1’	117.1	117.2	117.1	117.0
C10-C4-C3	121.9	121.8	122.0	122.1
C3-C1’=O	122.5	122.3	122.6	122.6
O=C1’-O	123.8	124.0	123.6	123.5
C3-C1’-O17	113.7	113.6	113.7	113.8
C1’-O-C2’	116.2	116.3	116.3	116.2
C4-C3-C2	120.3	120.3	120.3	120.3
C3-C2=O	128.2	128.5	128.0	128.2
C3-C2-O	115.7	115.6	115.7	115.6
C2-O-C9	124.0	124.0	123.9	123.9
O=C2-O	116.1	115.9	116.3	116.2
O-C9-C10	120.6	120.6	120.8	120.9
O-C9-C8	118.0	117.8	118.1	118.1
C9-C10-C4	117.5	117.6	117.2	117.1
C9-C8-C7	119.0	118.9	119.0	118.9
C8-C9-C10	121.4	121.7	121.1	121.0
C8-C7-C6	120.0	119.5	120.8	121.2
C7-C6-C5	121.4	122.2	120.1	119.8
C6-C5-C10	119.1	118.7	119.7	119.5
C5-C10-C4	123.3	123.4	123.4	123.2
C7-C6-Br	119.0			
C5-C7-Br	119.6			
C7-C6-N		118.9		
C5-C6-N		118.9		
C6-N-O		118.1		
C6-N-O		118.0		
O-N-O		123.9		
C7-C6-O			116.6	
C5-C6-O			123.3	
C7-C6-O				115.4
C5-C7-O				124.8
C7-O-Me				118.0
MAE	/	0.2	0.2	0.3

Table S7. Energies (in kJ mol^{-1}) of the second order pertubation theory stabilization interactions (at B3LYP-D3BJ/6-311++G(d,p) level of theory)

Type of interaction	**6-Br**	**6-NO2**	**6-OH**	**6-OCH3**
π(C3-C4)→π(C2-O)	109	107	108	97
π(C3-C4)→σ(C1’-O16)	45	53	45	/
π(C3-C4)→π(C1’-O)	83	80	84	/
π(C5-C10)→σ(C6-Br)	23	/	/	/
π(C5-C10)→σ(C6-N)	/	17	/	/
π(C5-C10)→σ(C6-O)	/	/	15	/
π(C5-C10)→σ(C6-O)	/	/	/	21
π(C9-C10)→π(C3-C4)	85-150	87-113	55-100	80-120
σ(C6-Br)→π(C5-C10)	13	/	/	/
σ(C6-Br)→π(C7-C8)	12	/	/	/
σ(C6-N)→π(C5-C10)	/	5	/	/
σ(C6-N)→π(C7-C8)	/	5	/	/
σ(C6-O)→π(C5-C10)	/	/	23	/
σ(C6-N)→π(C5-C10)	/	/	42	/
LP(O)→π(C2-O)	147	139	152	137
LP(O)→π(C9-C10)	124	129	116	67
LP(O)→π(C2-0)	70	70	68	69
LP(O)→π(C2-O)	150	156	146	146
LP(O)→π(C1’-O)	124	124	124	123
LP(O)→π(C3-C1’)	75	76	74	75
LP(Br)→π(C5-C6)	40	/	/	/
LP(Br)→π(C6-C7)	14	/	/	/
LP(O)→π(N-O)	/	540	/	/
LP(O)→π(C5-C6)	/	/	100	/
LP(O)→π(C6-C7)	/	/	5	/
LP(O)→π(C6-C7)	/	/	/	23

Table S8. AIM parameters (electron density/Laplacian) for the selected bonds (at B3LYP-D3BJ/6-311++G(d,p) level of theory)

Bond	6-Br	6-NO2	6-OH	6-OMe
C2−C3	0.2578/-0.7068	0.2759/-0.7086	0.2760/-0.7070	0.2764/-0.7084
C2=O	0.4199/-0.2263	0.4230/-0.1981	0.4173/-0.2473	0.4173/-02484
C2−O	0.2662/-0.4372	0.2611/-0.4438	0.2695/-0.4283	0.2687/-0.4286
C9−O	0.2801/-0.2302	0.2867/-0.2399	0.2758/-0.2127	0.2763/-0.2112
C8−C9	0.3133/-0.8952	0.3118/-0.8904	0.3128/-0.8906	0.3112/-0.8872

C7−C8	0.3109/-0.8685	0.3145/-0.8891	0.3122/-0.8741	0.3138/-0.8820
C7−C6	0.3049/-0.8401	0.3061/-0.8531	0.3064/-0.8598	0.3040/-0.8482
C6−Br	0.1561/-0.1435	/	/	/
C6-N	/	0.2591/-0.6619	/	/
N-O	/	0.4964/-1.0404	/	/
C6-O	/	/	0.2849/-0.3920	/
C6-O(CH_3)	/	/	/	0.2886/-0.3871
C6−C5	0.3164/-0.8923	0.3167/-0.9020	0.3162/-0.8940	
C5−C10	0.2999/-0.8161	0.3049/-0.8434	0.2988/-0.8081	0.3143/-0.8814
C9−C10	0.3092/-0.8708	0.3076/-0.8647	0.3096/-0.8704	0.3106/-0.8757
C4−C10	0.2926/-0.7994	0.2918/-0.7969	0.2931/-0.8018	0.2938/-0.8049
C3−C4	0.3292/-0.9568	0.3307/-0.9646	0.3281/-0.9507	0.3276/-0.9482
C3−C1’	0.2636/-0.6562	0.2629/-0.6533	0.2642/-0.6591	0.2643/-0.6587
C1’=O	0.4101/-0.2406	0.4109/-0.2350	0.4096/-0.2433	0.4094/-0.2460
C1’−O	0.3126/-0.4764	0.3136/-0.4761	03117/-0.4767	0.3116/-0.4769
C2’−O	0.2295/-0.2622	0.2288/-0.2562	0.2299/-0.2658	0.2301/-0.2674

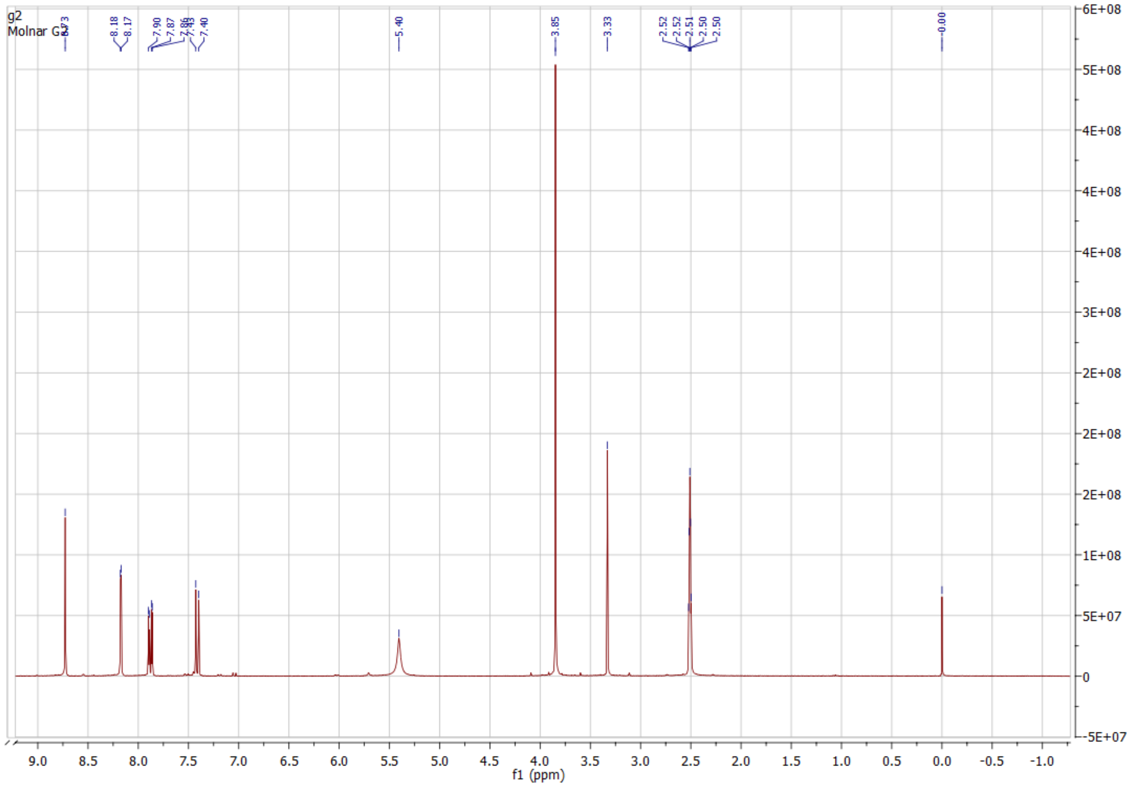

Figure S1. ^{1}H NMR spectrum of **6-Br**.

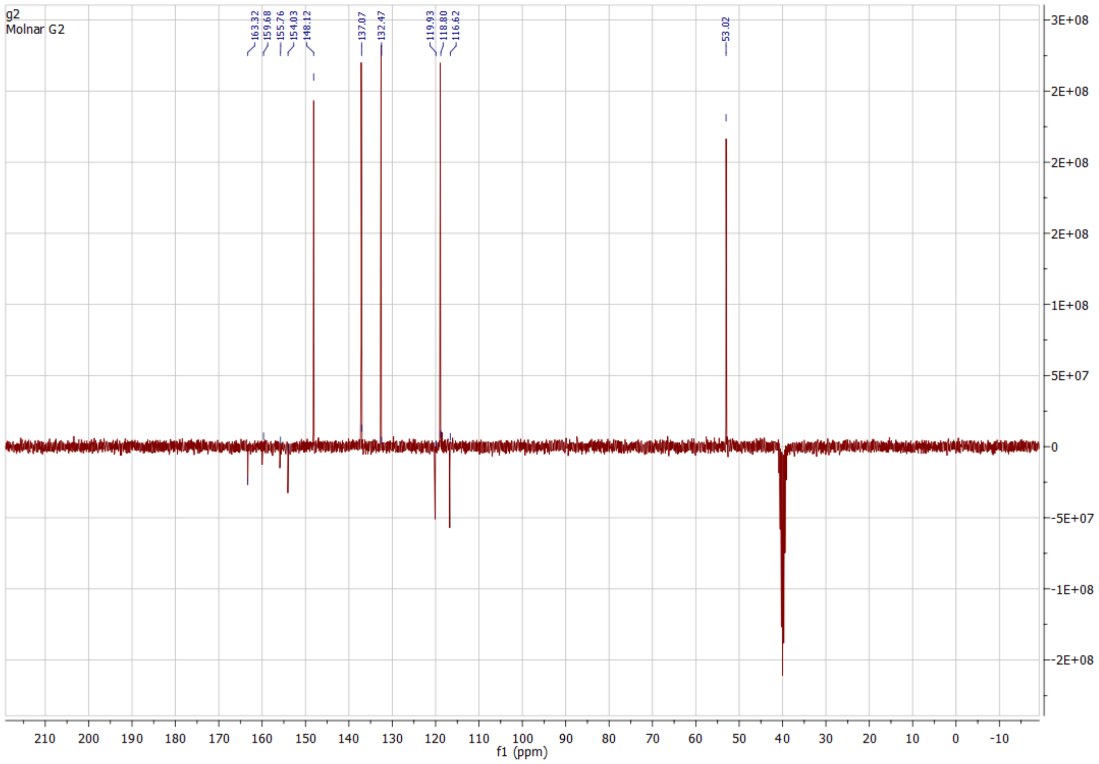

Figure S2. ^{13}C NMR spectrum of **6-Br**.

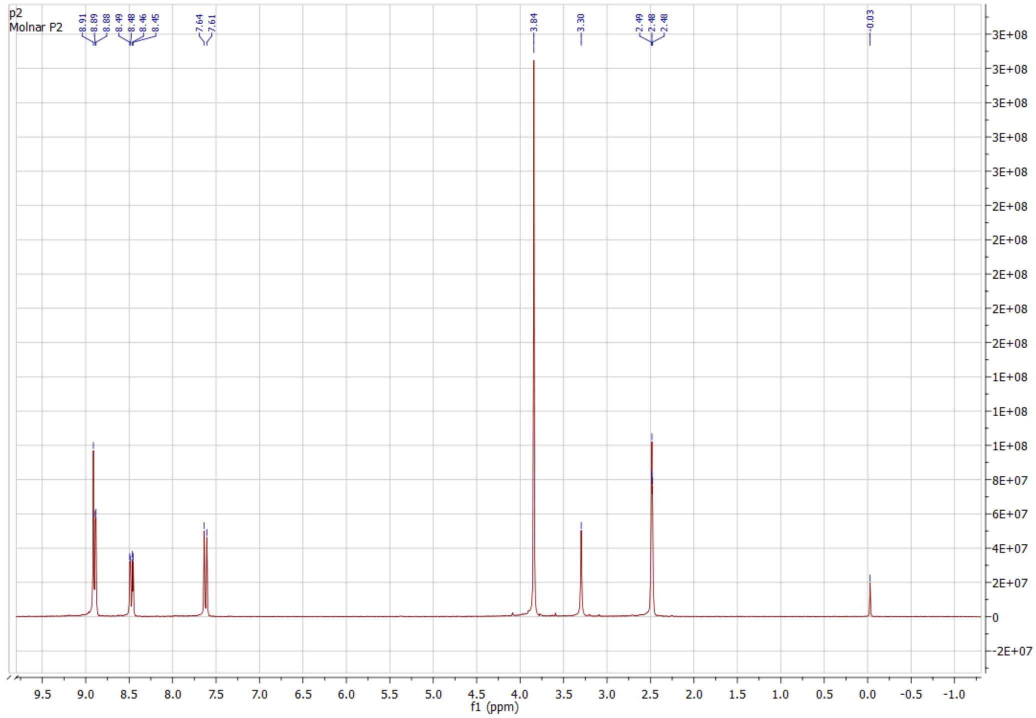

Figure S3. ^{1}H NMR spectrum of **6-NO2**.

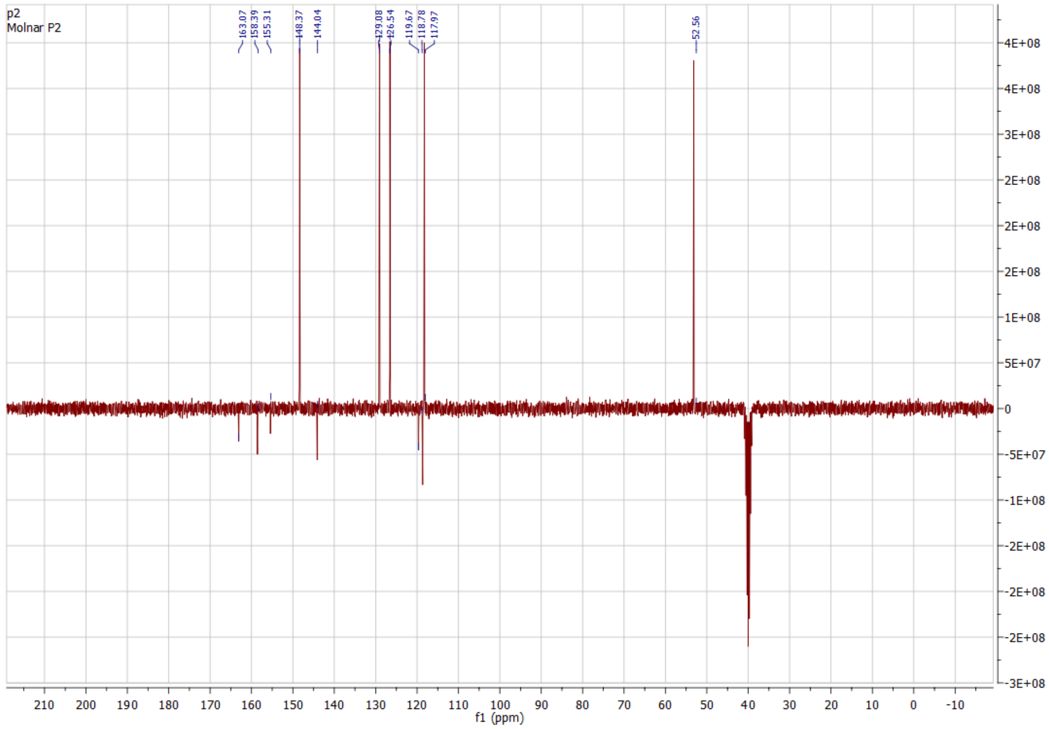

Figure S4. ^{13}C NMR spectrum of **6-NO2**.

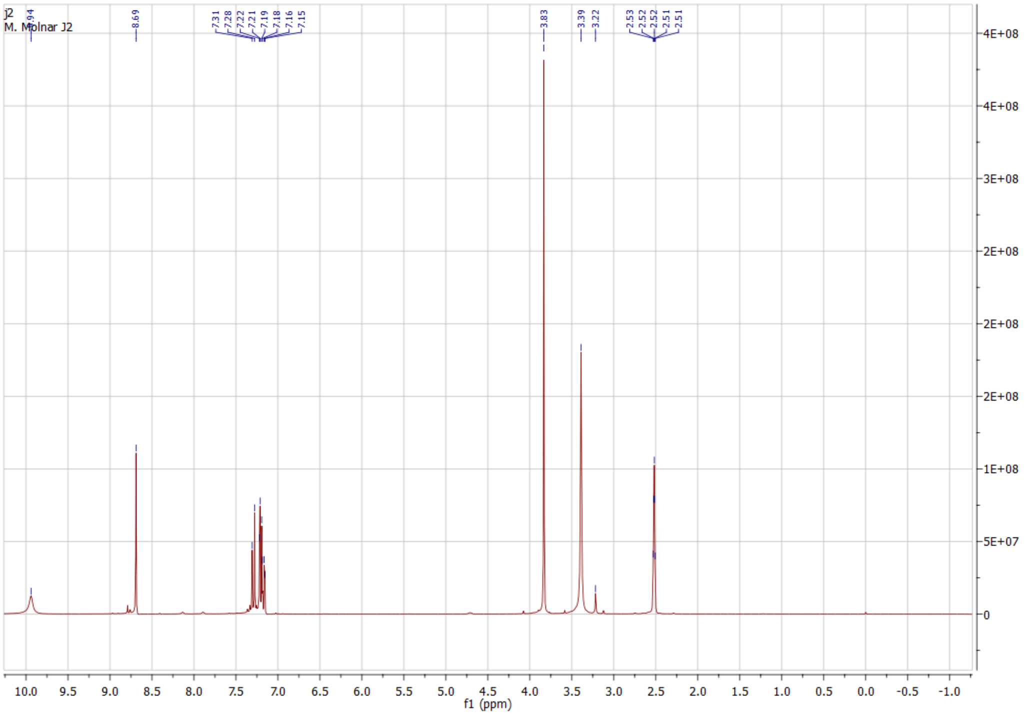

Figure S5. ^{1}H NMR spectrum of **6-OH**.

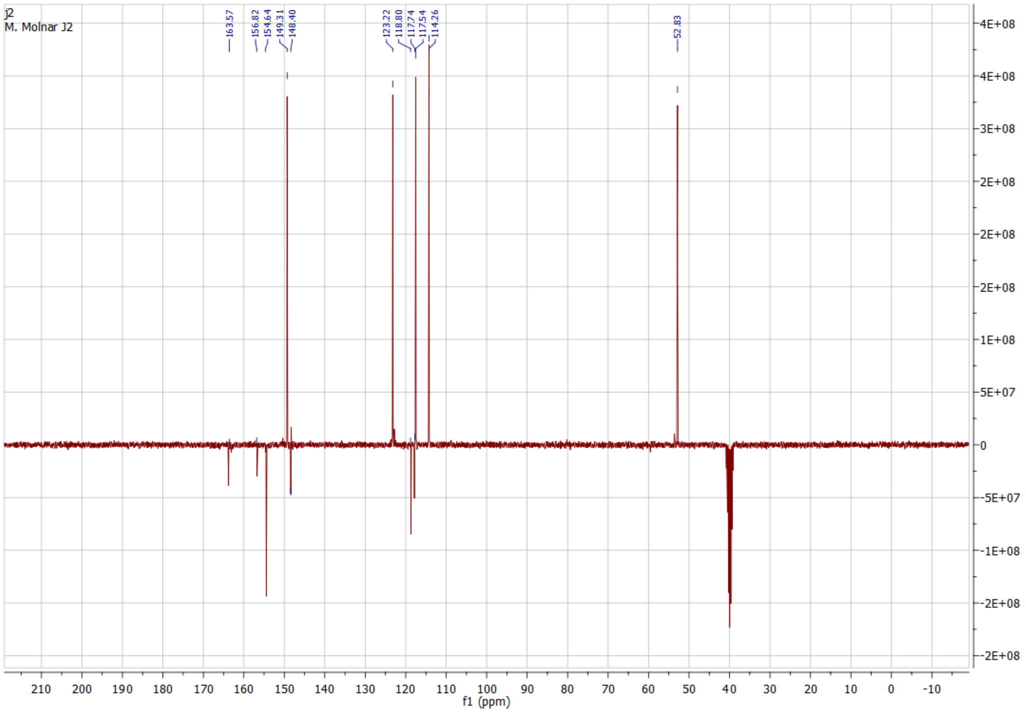

Figure S6. ^{13}C NMR spectrum of **6-OH**.

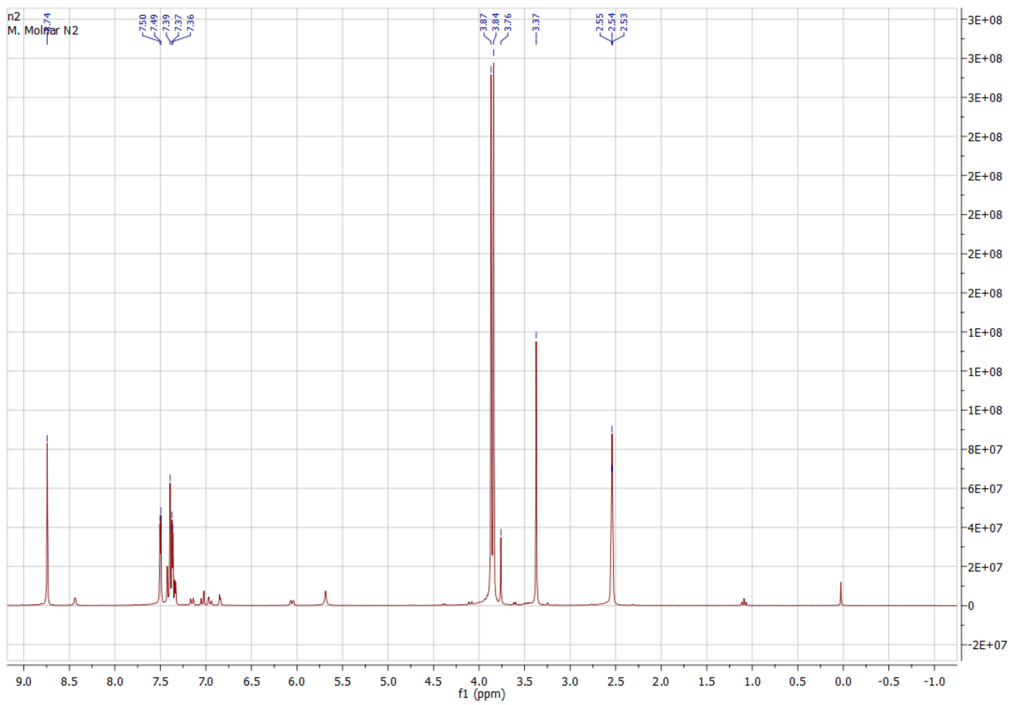

Figure S7. ^{1}H NMR spectrum of **6-OMe**.

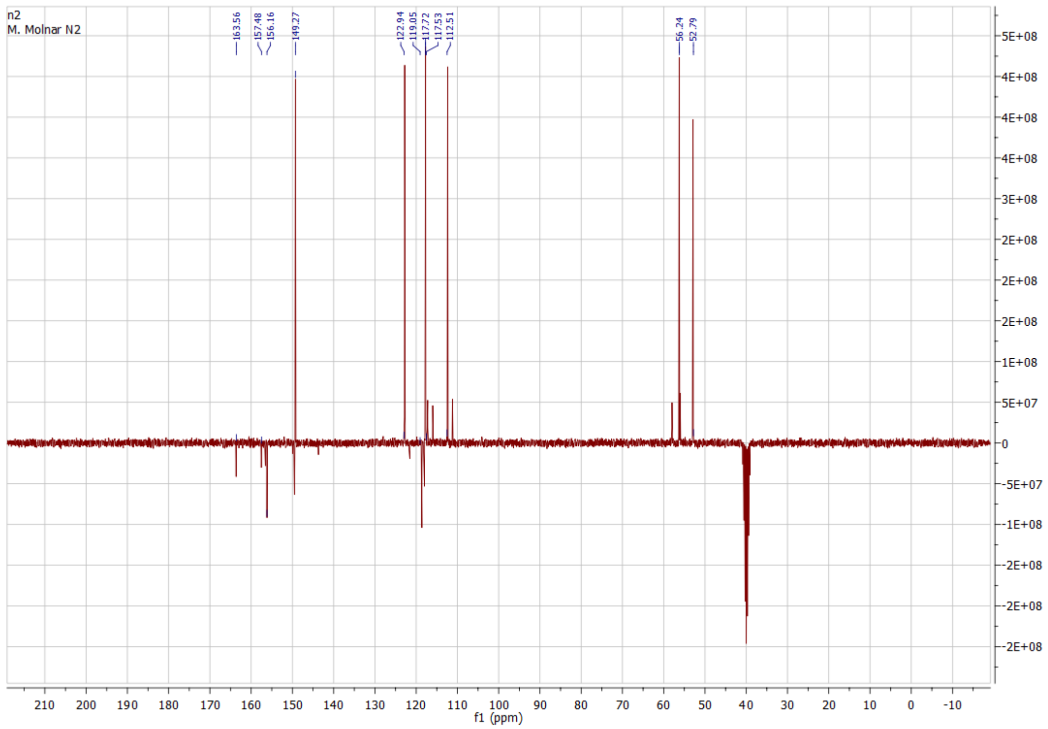

Figure S8. ^{13}C NMR spectrum of **6-OMe**.

Table S9. Experimental and theoretical (at B3LYP-D3BJ/6-311++G(d,p)) ^{1}H and ^{13}C chemical shifts of **6-NO2**

^{1}H chemical shifts [ppm]			^{13}C chemical shifts [ppm]		
H atom	Experimental	Theoretical	C atom	Experimental	Theoretical
C2'-H (3)	3.94	3.80	C2'	53	55
C8-H	7.58	7.61	C3	118	119
C7-H	8.86	8.89	C8	119	119
C5-H	8.79	8.48	C10	120	119
C4-H	8.86	8.91	C5	127	129
R		0.999	C6	129	132
MAE [ppm]		0.14	C7	144	145
			C4	148	153
			C2	155	156
			C9	158	162
			C1'	163	166
			R		0.995
			MAE [ppm]		2

Table S10. Experimental and theoretical (at B3LYP-D3BJ/6-311++G(d,p)) ^{1}H and ^{13}C chemical shifts of **6-OH**

^{1}H chemical shifts [ppm]			^{13}C chemical shifts [ppm]		
H atom	Experimental	Theoretical	C atom	Experimental	Theoretical
C2'-H (3)	3.82	3.83	C2'	53	55
C8-H	7.45	7.22	C3	114	113
C7-H	7.52	7.30	C8	118	118
C5-H	7.05	7.15	C10	118	119
C4-H	8.79	8.69	C5	119	120
R		0.999	C6	132	125
MAE [ppm]		0.13	C7	148	153
			C4	149	153
			C2	154	155
			C9	157	158
			C2'	164	167
			R		0.996
			MAE [ppm]		2

Table S11. Experimental and theoretical (at B3LYP-D3BJ/6-311++G(d,p)) ^{1}H and ^{13}C chemical shifts of **6-OMe**

^{1}H chemical shifts [ppm]			^{13}C chemical shifts [ppm]		
H atom	Experimental	Theoretical	C atom	Experimental	Theoretical
C2'-H (3)	3.84	3.85	C2'	53	55
OCH_3	3.76	3.84	C3	56	56
C8-H	7.40	7.50	C8	113	108
C7-H	7.50	7.68	C10	118	117
C5-H	7.00	6.90	C5	118	119
C4-H	8.74	8.87	C6	119	120
R		0.999	C7	123	129
MAE [ppm]		0.10	C4	149	153
			C2	149	153
			C9	156	158
			C1'	157	158
			OCH_3	164	167
			R		0.997
			MAE [ppm]		2

Table S12. Experimental and theoretical electronic transitions of investigated coumarin derivatives

Compound	Experimental wavelength [nm]	Calculated wavelength [nm]	Transition with the probability	Oscillatory strenght
**6-Br**	347	351	HOMO→LUMO (96%)	0.1344
	291	298	HOMO-1→LUMO (85%)	0.4008
	228	230	HOMO→LUMO+1 (38%) HOMO→LUMO+2(45%)	0.4658
**6-NO2**	331	333	HOMO→LUMO (81%)	0.1687
	271	300	HOMO→LUMO+1 (88%)	0.3629
		287	HOMO-1→LUMO (40%) HOMO-2→LUMO (49%)	0.1250
		280	HOMO-3→LUMO (33%)	0.1878
		275	HOMO-3→LUMO (50%)	0.1325
**6-OH**	300	301	HOMO→LUMO (97%)	0.1269

	368	377	HOMO-1→LUMO (92%)	0.3989
**6-OCH3**	365	385	HOMO→LUMO (98%)	0.1301
	296	301	HOMO-1→LUMO (93%)	0.4093
	229	239	HOMO→LUMO+1 (63%)	0.2813
	202	212	HOMO-1→LUMO+1 (51%)	0.3896

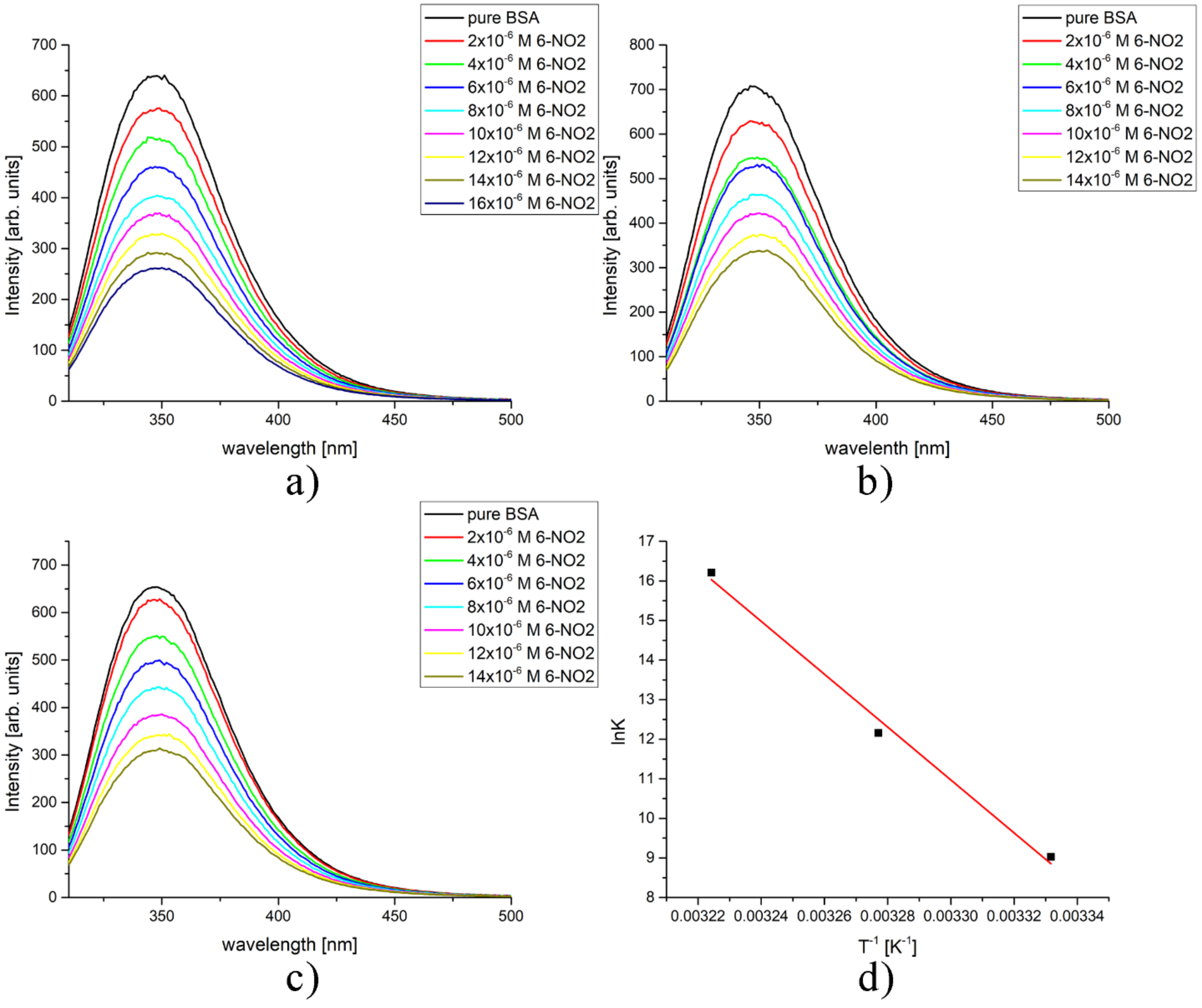

Figure S9. The BSA-binding fluorescence curves for **6-NO2** at a) 27°C, b) 32°C, and c) 37°C, and d) Van't Hoff's plot for binding

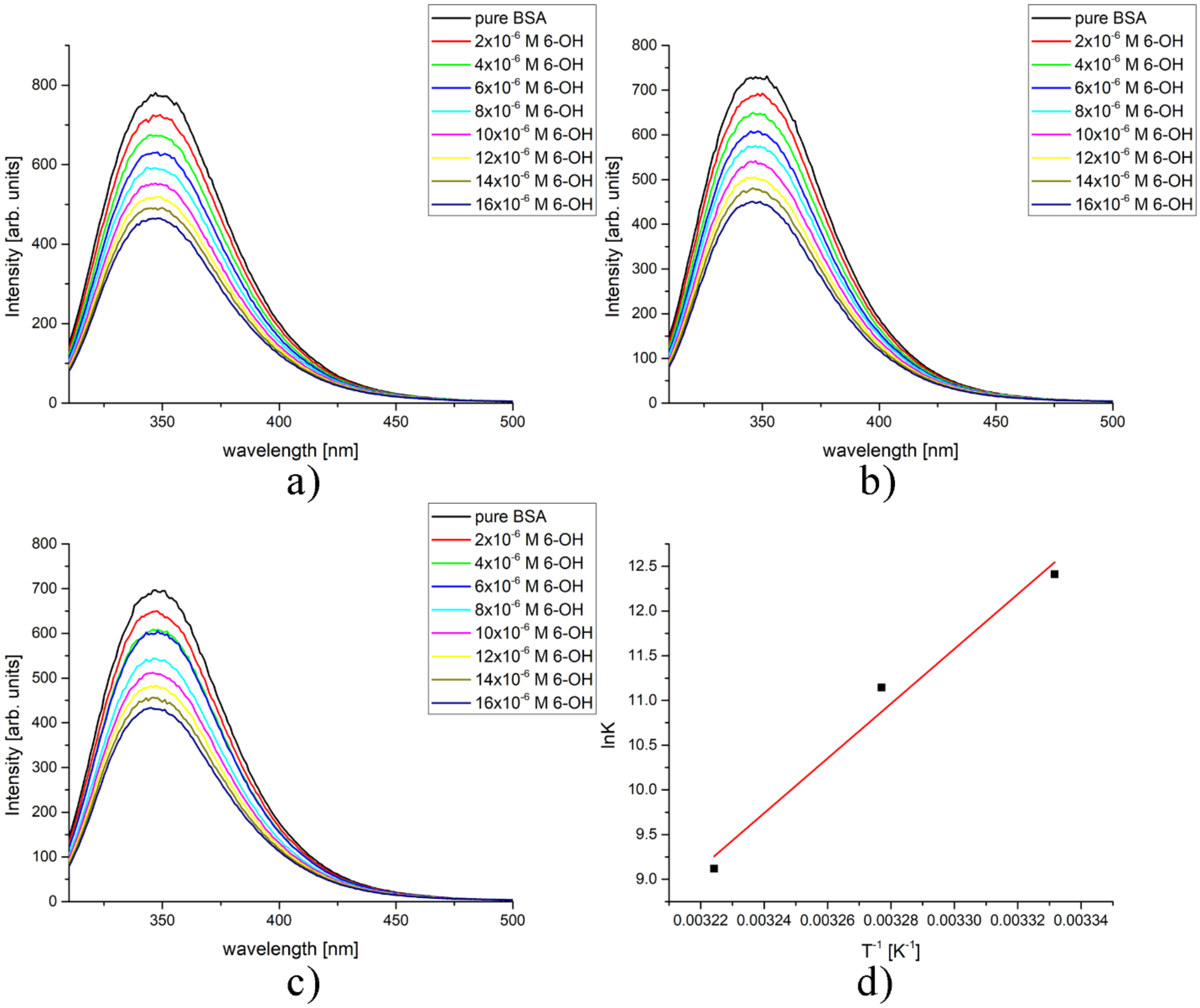

Figure S10. The BSA-binding fluorescence curves for **6-OH** at a) 27ºC, b) 32ºC, and c) 37ºC, and d) Van't Hoff's plot for binding

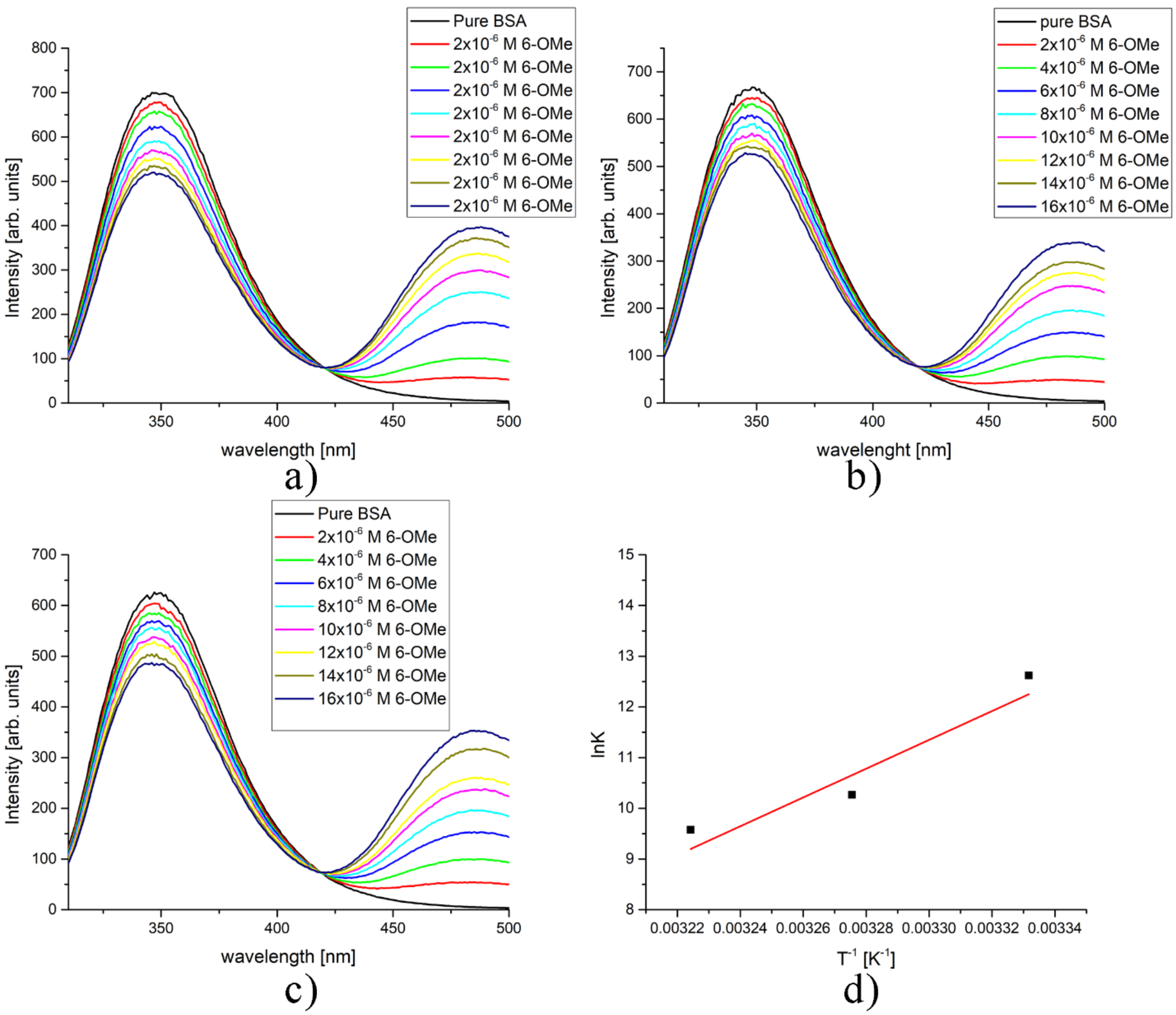

Figure S11. The BSA-binding fluorescence curves for **6-OCH$_3$** at a) 27°C, b) 32°C, and c) 37°C, and d) Van't Hoff's plot for binding

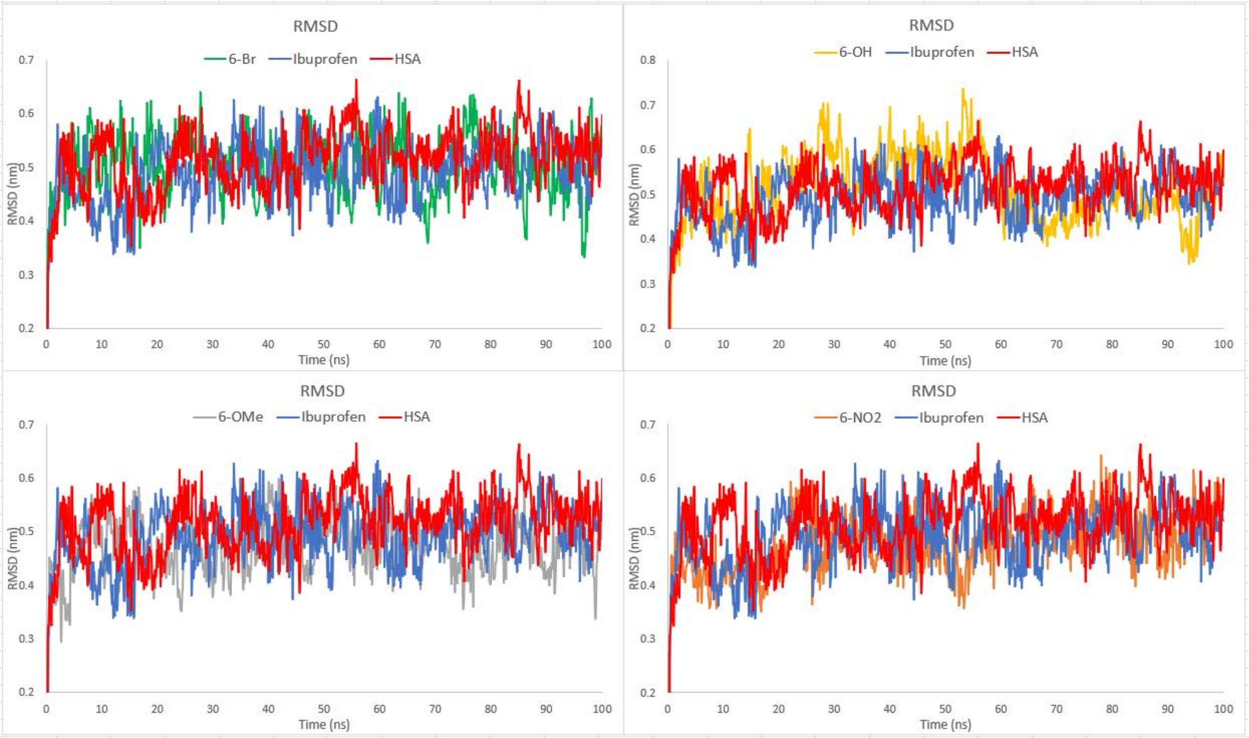

Figure S12. RMSD diagrams describing interactions of investigated compounds with HSA in comparison to ibuprofen.
